# DNA methylation signatures in skeletal muscle associated with physical function in healthy older adults

**DOI:** 10.1007/s11357-026-02410-9

**Published:** 2026-08-08

**Authors:** Xiaoxiao Wen, Mingjing Chen, Guanhong Miao, Christopher Wu, Anna Picca, Javier A. Tamargo, Xiangyang Lou, Kevin Wu, Stephen Anton, Christiaan Leeuwenburgh, Jinying Zhao

**Affiliations:** 1 Health Informatics Institute, University of South Florida, 3650 Spectrum Boulevard, Tampa, FL 33612 USA; 2 Department of Physiology and Aging, College of Medicine, University of Florida, Gainesville, FL USA; 3 Department of Medicine and Surgery, LUM University, Casamassima, Italy; 4 Fondazione Policlinico Universitario “Agostino Gemelli” IRCCS, Rome, 00168 Italy; 5 Department of Community Dentistry and Behavioral Science, University of Florida, Gainesville, FL USA; 6 Department of Biostatistics, University of Florida, Gainesville, FL USA; 7 Department of Medicine, Division of Geriatric Medicine, College of Medicine, Institute On Aging, University of Florida, Gainesville, FL USA

**Keywords:** Epigenetics, DNA methylation, Skeletal muscle, Physical function, Healthy aging

## Abstract

Despite the substantial variability in physical function among older adults, the molecular mechanisms remain poorly characterized, particularly within skeletal muscle. This study aimed to determine the patterns of DNA methylation in skeletal muscle associated with physical function in healthy older adults. We analyzed DNA methylation (EPIC v2 array; 875,554 CpG sites) in skeletal muscle from 92 healthy older adults (median age 74; 62% female). Associations were examined across five phenotypes: Short Physical Performance Battery (SPPB), 6-min walk test (6MWT), handgrip strength, perceived disability (PAT-D), and lifestyle health (modified Life’s Essential 8). Linear regression models adjusted for age, sex, race, BMI, and muscle fiber composition. Genomic inflation corrected via the BACON method (FDR < 0.05). Gene set enrichment analysis was performed on suggestive hits (FDR < 0.1). We identified significant differentially methylated probes (DMPs) and regions (DMRs) across all phenotypes: SPPB (70 DMPs, 22 DMRs), 6MWT (16 DMPs, 566 DMRs), handgrip strength (2 DMRs), PAT-D (19 DMPs, 1 DMR), and lifestyle health (2 DMPs). DMRs largely overlapped promoters. Identified genes overlapped known musculoskeletal and neurological GWAS hits, including *RUNX2* and *FOXL1* (bone mineral density*)*, *IGFBP3* (muscle mass), and *NEK1* and *SHANK1* (neurological function). Enrichment analysis revealed that 6MWT-associated genes relate to nervous and skeletal system development, while handgrip-associated genes involve cytoskeletal dynamics and protein assembly. Epigenetic variation in aging skeletal muscle is associated with physical function. The enrichment of pathways related to nervous and musculoskeletal development suggests specific epigenetic mechanisms underlying functional decline, offering potential targets for intervention in older adults.

**Graphical abstract:**

**Figure:**
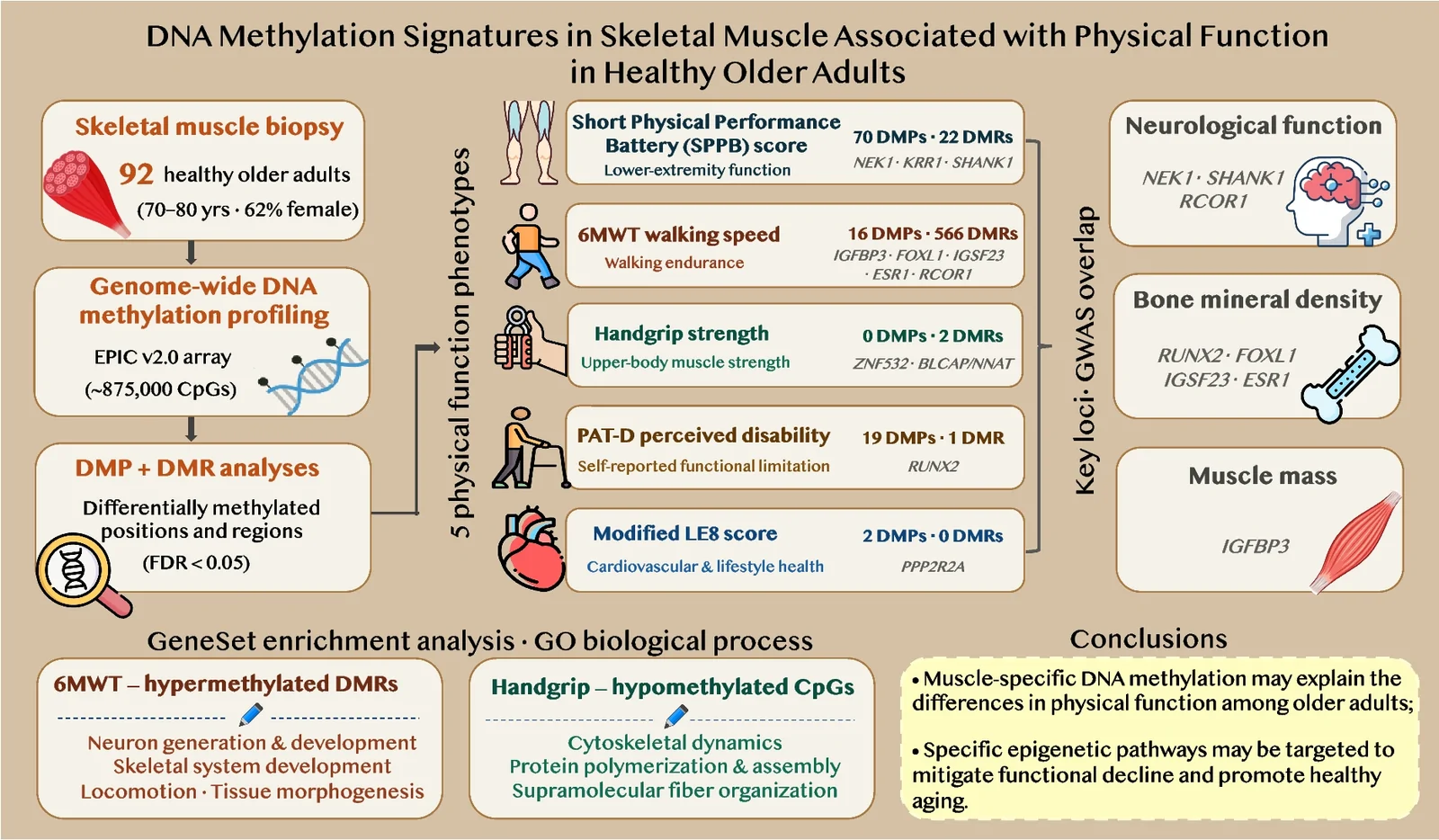

**Supplementary information:**

The online version contains supplementary material available at https://doi.org/10.1007/s11357-026-02410-9.

## Introduction

Mobility limitations represent a critical public health challenge for the aging population. In the USA, over three million adults aged 65 and older report walking difficulties, which is projected to reach 5.5 million by 2050 [1]. Declining physical function is a strong predictor of falls, hospitalization, loss of independence, and mortality [2–5]. Furthermore, aging is characterized by striking interindividual variability, while some individuals maintain high levels of mobility into their ninth decade, others experience early and rapid declines in strength and endurance [6]. This heterogeneity suggests that chronological age is an insufficient marker for physical vitality. Even among otherwise healthy individuals, age-related impairments in mobility and endurance often emerge gradually, preceding overt clinical disability. Despite the profound societal and individual burden, the biological mechanisms driving this significant variability in physical performance during aging remain incompletely understood.

Epigenetic alterations are recognized as a key hallmark of aging and reflect the cumulative influence of genetics, environmental exposures, and physiological stressors across the lifespan [7]. DNA methylation, in particular, offers a promising molecular framework for understanding age-related functional decline. While alterations in DNA methylation have been associated with inter-individual variability in physical disability and functional impairment [8–10], most evidence was derived from whole blood, which may fail to capture the molecular processes occurring within skeletal muscle, the tissue directly responsible for locomotion and strength. To our knowledge, two prior blood-based epigenome-wide association studies (EWAS) [8, 11] found no significant associations with handgrip strength or walking speed, potentially because epigenetic changes are tissue-specific. Moreover, previous studies may be confounded by age-related comorbidities, masking the direct relationship between methylation and physical function. In addition, although an earlier study in postmitotic muscle tissue identified 500 CpG sites [12], the study included only 48 male participants and a focus on chronological age rather than functional phenotypes. Consequently, the relationship between muscle DNA methylation and objective physical performance in older adults remains largely unexplored.

The current study addresses these gaps by evaluating the association between skeletal muscle DNA methylation and a comprehensive battery of physical function measures in healthy older adults, independent of established risk factors. To capture broader epigenetic signatures, we complemented our site-specific analysis with a regional approach (differentially methylated region analysis) to identify coordinated DNA methylation changes associated with physical performance. Furthermore, we employed pathway analysis to characterize the biological mechanisms linking these epigenetic features to physical performance.

## Methods

### Study design and population

The “Muscle Iron Flow Study” is an ongoing longitudinal cohort study in Florida, USA, investigating the relationship between iron regulation, mitochondrial function, and physical performance in older adults. Recruitment and baseline assessments were completed in December 2024, and participants are being followed annually for a total of 3 years, with study completion anticipated in 2027. The study protocol includes annual assessments of physical function, cardiometabolic and lifestyle factors, and biological markers related to iron regulation and mitochondrial function. Blood samples and physical function assessments are collected annually, while skeletal muscle biopsies were obtained at baseline and scheduled to be repeated at the three-year follow-up visit.

**Fig. 1:**
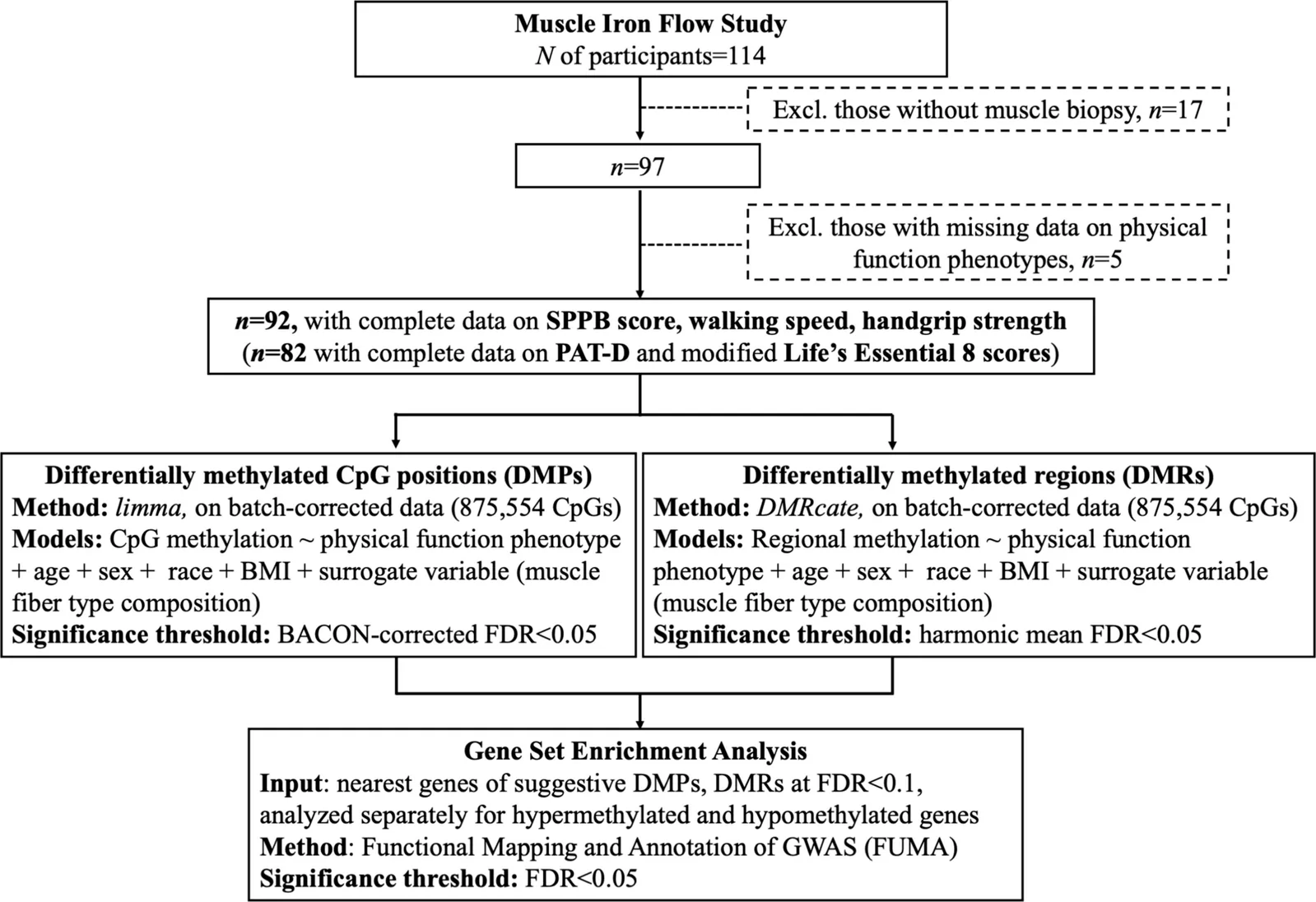
Flowchart of participant selection and statistical analysis

The flowchart of participant inclusion and exclusion is shown in Fig. 1. Eligible participants were community-dwelling healthy older adults aged 70–80 years at enrollment. Exclusion criteria included conditions or treatments that could substantially impair mobility or interfere with study participation. Briefly, these included major cardiovascular, pulmonary, neurologic, psychiatric, inflammatory, gastrointestinal, metabolic (including diabetes), hematologic, hepatic, or renal disease; recent cancer, fracture, surgery, or joint replacement; neuromuscular disorders; severe osteoarthritis; substance abuse, recent smoking, or excessive alcohol use; and contraindications to muscle biopsy (e.g., lidocaine allergy, anticoagulant or anabolic hormone use, inability to pause antiplatelet therapy). More details about the study population and inclusion criteria are provided in the Supplemental Methods.

For the present analyses, 97 participants provided skeletal muscle biopsies for DNA methylation analysis at baseline. Among them, 92 participants (median age, 74 years; female, 62%; 88% White) had data on physical function phenotypes at baseline and were included in the current analysis.

The study was approved by the Institutional Review Board of the University of Florida (IRB number: 201300725), and all participants provided written informed consent prior to participation.

### Assessment of physical function

Physical function was assessed using a comprehensive battery of validated measures capturing five functional domains: lower-extremity function, walking speed, upper-body muscle strength, self-perceived disability, and cardiovascular and lifestyle health. All physical function measures were analyzed as continuous outcomes. Specifically:

Lower-extremity function was evaluated using the Short Physical Performance Battery (SPPB) [13], which integrates performance on a 4-m walk, repeated chair stand test, and standing balance tasks, yielding a total score ranging from 0 to 12, with higher scores indicating better overall physical performance.

Walking speed was derived from the six-minute walk test (6MWT) [14], calculated as total distance walked (meters) divided by 360 s, reflecting walking endurance and overall functional capacity. The high reliability of the distance measured in a 6MWT suggests that even small changes in distance can reflect meaningful differences in functional capacity [15].

Upper-body muscle strength was measured using handgrip strength, a widely used index of upper body strength that correlates with total body strength [16, 17]. Handgrip strength was assessed with a handheld dynamometer (Jamar) as the average of three readings. We considered the highest measurement from either the dominant or nondominant hand as the final measure.

Self-perceived disability was assessed using the Pepper Assessment Tool for Disability (PAT-D) [18], a 19-item questionnaire evaluating mobility, activities of daily living, and instrumental activities of daily living, with total scores ranging from 1 to 5 and higher values indicating greater perceived functional limitation.

Cardiovascular and lifestyle health was summarized using a modified Life’s Essential 8 (LE8) score. The modified LE8 score was calculated as the mean of the six available components (blood pressure, blood glucose, BMI, diet quality, physical activity, and sleep), each scaled from 0 to 100. We did not include nicotine exposure and blood lipid in the calculation due to unavailability of data. Consistent with the original LE8 definition, the resulting score ranged from 0 to 100, with higher scores indicating better cardiovascular and lifestyle health. Detailed scoring procedures for individual components are provided in the Supplemental Methods. Briefly, resting blood pressure was measured using standardized clinical procedures, and fasting plasma glucose concentrations were obtained from blood samples collected after an overnight fast. Physical activity levels were assessed using the Community Healthy Activities Model Program for Seniors (CHAMPS) questionnaire, which captures the frequency and duration of common activities in older adults. Moderate-to-vigorous physical activity was estimated by summing weekly minutes spent in activities of moderate or vigorous intensity. Sleep duration was self-reported as a component of the Pittsburgh Sleep Quality Index. Dietary intake was assessed using the total Healthy Eating Index 2015 score, a validated measure of adherence to the Dietary Guidelines for Americans.

### Assessment of covariates

Demographic characteristics, including age, sex, and race, were collected at enrollment using standardized questionnaires. Anthropometric and clinical measures were obtained at baseline following standardized protocols. Body weight and height were measured with participants wearing light clothing and no shoes, and BMI was calculated as weight in kilograms divided by height in meters squared.

### Skeletal muscle biopsy and DNA extraction

Skeletal muscle tissue was obtained at baseline from the vastus lateralis of the dominant thigh (unless otherwise indicated by the participant) using a percutaneous biopsy technique under local anesthesia. Approximately 150–500 mg of muscle tissue was collected per participant, immediately processed and stored at − 80 °C until analysis. Genomic DNA was extracted using QIAGEN DNeasy Blood & Tissue Kit (catalog number: 69504) according to the manufacturer’s protocol. A detailed protocol for DNA extraction was provided in Supplemental Methods.

### DNA methylation profiling, quality control, and batch correction

Epigenome-wide DNA methylation profiling was performed using the Infinium MethylationEPIC v2.0 BeadChip (Illumina, San Diego, CA, USA) following the manufacturer’s protocol, which interrogates 930,075 CpG sites across the human genome.

Raw intensity data were imported into R, and pre-processing, quality control (QC), and normalization of the methylation data were performed using R package *minfi *[19] and *wateRmelon *[20]. We removed probes that had > 1% of samples with detection *P* > 0.05, had < 3 beads in ≥ 5% of samples, mapped to sex chromosomes, contained SNPs with minor allele frequency (MAF) ≥ 0.05, belong to previously reported cross-reactive probes, or were non-CpG sites. Beta Mixture Quantile (BMIQ) normalization was applied to correct for probe-type bias. Detailed QC procedures were provided in Supplemental Methods. Following QC, a total of 875,554 CpG sites and 92 samples were retained for downstream analyses. We used methylation *M*-values for all statistical association and region-based analyses. Prior to the analyses, batch effect in the methylation data was corrected using the ComBat function in R package *sva* [21]. Briefly, ComBat is an empirical Bayes method that removes systematic technical variation attributable to known batch effects while preserving biological variation of interest.

### Statistical analysis

Clinical characteristics of study participants were summarized with continuous measures expressed as mean ± standard deviation (SD) or median with interquartile range as appropriate and categorical measures as frequency with percentage. Statistical analyses were performed using R 4.5.1 software. To maximize the use of available data and statistical power, we used data from 92 participants for the SPPB, 6MWT, and handgrip analyses, and data from 82 participants for the PAT-D and LE8 score analyses. Figure 1 shows the flowchart of participant selection and analytic plan.

### Identification of differentially methylated positions (DMPs)

We examined differentially methylated positions (DMPs) in skeletal muscle associated with each physical function phenotype (i.e., SPPB score, 6MWT walking speed, handgrip strength, PAT-D total score, and LE8 score), separately. Briefly, DMPs were detected by linear regression using the R package *limma* [22]. The model included methylation *M*-values as the dependent variable and each physical function outcome modeled as the independent variable, adjusting for age, sex, race, and BMI. To account for potential confounding from muscle fiber-type composition and other unmeasured sources of systematic variation, we incorporated a surrogate variable generated via the *sva* R package [21] into our models. We utilized this reference-free approach because muscle fiber-type composition was not directly measured, and validated muscle fiber-type methylation reference panels are currently unavailable. To enhance the stability of variance estimates, we applied empirical Bayes moderation to the fitted models using the eBayes function from the *limma* package.

Following the DMP analysis, genomic inflation was assessed and corrected with the BACON method implemented in the R package *bacon* [23]. Briefly, BACON is a Bayesian approach specifically developed for epigenome-wide association studies to estimate and correct bias and inflation in test statistics, addressing the tendency of conventional genomic inflation factors (*λ*) to overestimate true inflation [23]. We applied BACON to obtain inflation-corrected effect estimates, standard errors, and p-values for each of the CpG-level associations. Then, we generated quantile–quantile (Q–Q) plots using the corrected *p*-values and calculated corrected genomic inflation factors (*λ*) to evaluate residual inflation.

To account for multiple testing, we applied the Benjamini–Hochberg procedure to the BACON-corrected *p*-values. Statistical significance was defined as false discovery rate (FDR) < 0.05.

### Identification of differentially methylated regions (DMRs)

We performed differentially methylated region (DMR) analyses using the R package *DMRcate *[24] to identify genomic regions where coordinated methylation changes were associated with physical function outcomes. The model adjusted for age, sex, race, BMI, and a latent surrogate variable.

Regions were defined as significant based on a harmonic mean FDR < 0.05 and the presence of at least three significantly associated CpG sites within a 1000 bp window. For each DMR, the effect size was calculated as the mean methylation change across all constituent CpGs per unit change in functional phenotype. Finally, DMRs were annotated to genomic features by identifying overlaps between DMRs and CpG sites using the findOverlaps function from the *GenomicRanges* package [25].

### Gene-set enrichment analysis

Significant DMPs and DMRs were mapped to the human genome using the Illumina EPICv2 annotation reference (IlluminaHumanMethylationEPICv2anno.20a1.hg38). We categorized CpG sites by genomic context (island, shore, shelf, or open sea) and gene-region annotation (promoter, 5′UTR, first exon, gene body, or 3′UTR) based on the hg38 reference genome.

We then performed gene-set enrichment analyses (GSEA) to investigate biological pathways on genes harboring DMPs and DMRs. To enhance detection sensitivity, we included gene sets with suggestive associations (FDR < 0.1). We analyzed hypermethylated and hypomethylated CpGs separately to capture direction-specific pathways. Functional annotation was conducted using the Functional Mapping and Annotation of GWAS (FUMA) GENE2FUNC module [26], utilizing all analyzed CpGs as the background set. Gene expression reference profiles were derived from the Genotype-Tissue Expression (GTEx) version 8 database across 54 tissue types and 30 general tissue types. We prioritized pathways from the KEGG and Gene Ontology (GO) databases, defining statistical significance at an FDR < 0.05 after excluding gene sets with fewer than two overlapping genes.

## Results

### Baseline characteristics

Of the 92 participants, 57 (62%) were female. The cohort had a median age of 74 years and a median BMI of 28.9 kg/m^2^ (Table 1). The majority of participants were White (88%). While the median SPPB score was approximately 11 for both males and females, males exhibited significantly higher walking speed based on 6MWT (1.2 vs. 1.0 m/s, *P* = 0.001) and handgrip strength (median 40.5 vs. 24.5 kg, *P* < 0.001) than females. Conversely, females reported slightly higher perceived disability, indicated by PAT-D total scores (1.2 vs. 1.0, *P* = 0.027). Finally, cardiovascular and lifestyle health, measured by Life’s Essential 8 (LE8), were comparable between sexes, averaging 67.4 for males and 62.9 for females.

**Table 1:** Baseline characteristics of study participants (*n* = 92)

Characteristics	All (*N* = 92)	Male (*n* = 35)	Female (*n* = 57)	*P*
Age, years	74.0 [71.0, 76.0]	74.0 [73.0, 76.0]	73.0 [71.0, 76.0]	0.083
BMI, (kg/m^2^)	28.9 [25.8, 31.6]	28.3 [26.3, 30.4]	29.3 [25.0, 32.0]	0.837
Race, *n* (%)				0.335
White	81 (88.0)	31 (88.6)	50 (87.7)	
Black	6 (6.5)	1 (2.9)	5 (8.8)	
Other	5 (5.4)	3 (8.6)	2 (3.5)	
SPPB score	11.0 [8.8, 12.0]	11.0 [9.0, 12.0]	11.0 [8.0, 12.0]	0.286
6MWT walking speed, m/s	1.1 (0.3)	1.2 (0.2)	1.0 (0.3)	0.001
Handgrip strength (kg)	28.2 [22.4, 39.0]	40.5 [35.8, 48.0]	24.5 [20.5, 28.0]	< 0.001
PAT-D total core	1.1 [1.0, 1.4]	1.0 [1.0, 1.2]	1.2 [1.0, 1.4]	0.027
Life’s Essential 8 score	64.6 (14.5)	67.4 (12.9)	62.9 (15.2)	0.151

Categorical variables were expressed as frequency (percentage) and continuous variables as median [Q25, Q75], except walking speed and Life’s Essential 8 score as mean (SD). Life’s Essential 8 score was modified based on availability of data (see “Methods” section for details). Between-group *P* values were calculated using two-sample *t* tests (for walking speed and Life’s Essential 8 score) or Wilcoxon rank-sum tests (for other non–normally distributed variables). Categorical variables were compared using *χ*^2^ tests or Fisher’s exact tests, as appropriate

*SPPB* short physical performance battery, *6MWT* 6-min walk test, *PAT-D* pepper assessment tool for disability

### Muscle DNA methylation associated with physical function measures

#### Associations with SPPB score

**Fig. 2:**
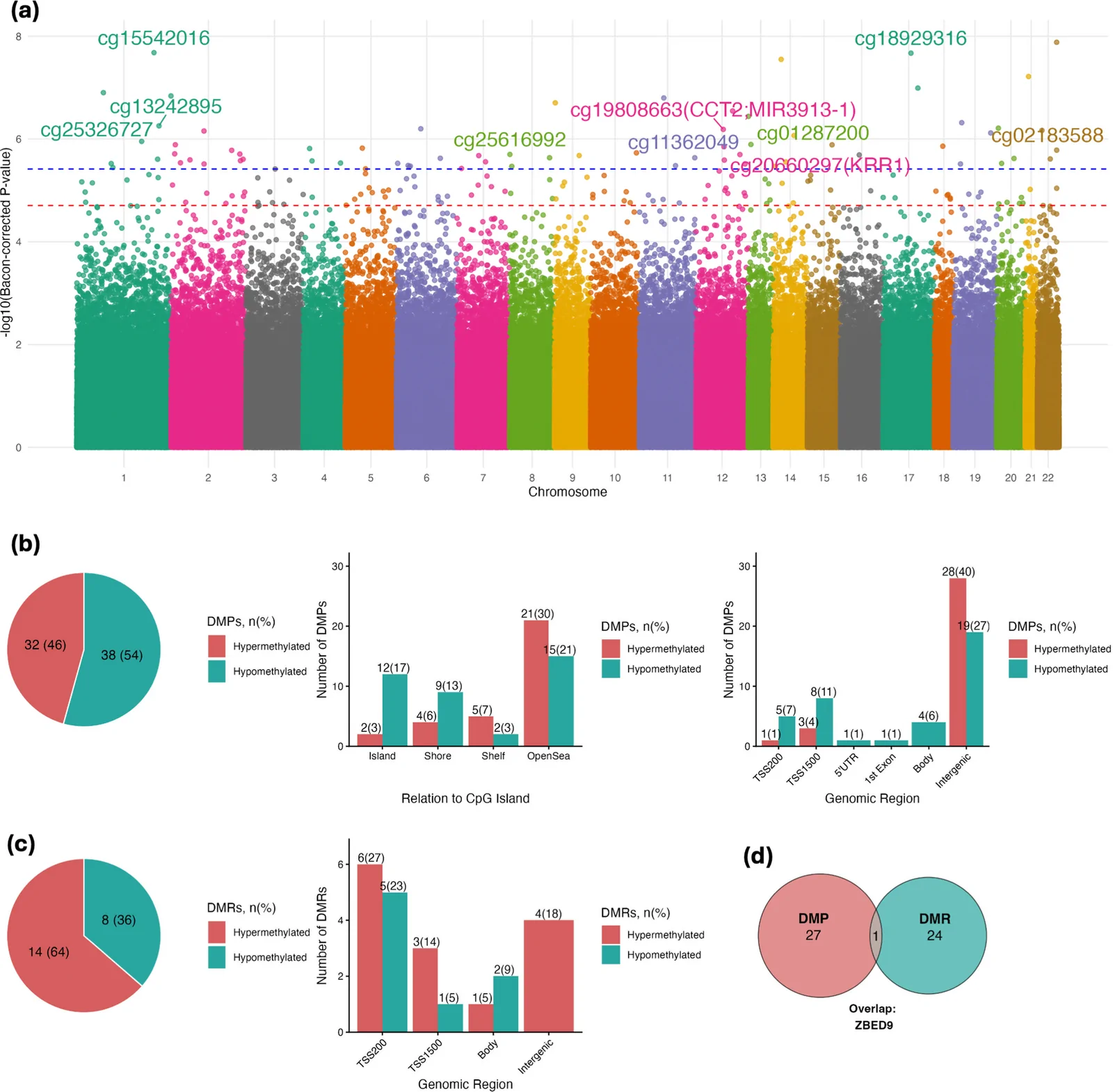
Differentially methylated positions (DMPs) and regions (DMRs) in human skeletal muscle associated with SPPB score. **a** Manhattan plots showing DMPs associated with SPPB score. Labelled are top 10 significant DMPs (five hypermethylated and five hypomethylated), ranked by beta coefficients. The dashed blue line indicates the statistical significance threshold (BACON-corrected FDR < 0.05), while the red line represents the suggestive significance threshold (BACON-corrected FDR < 0.1). **b** Annotations of significant DMPs by CpG island context and genomic context, with hypermethylated in red and hypomethylated in teal. **c** Annotations of significant DMRs by genomic context, with hypermethylated in red and hypomethylated in teal. **d** Venn diagram showing the number of differentially methylated genes mapped from DMPs and DMRs

In total, 70 DMPs were significantly associated with SPPB score at FDR < 0.05 (Fig. 2; Table 2; Supplemental Table S1). Of these, 46% were hypermethylated (i.e., higher methylation level associated with higher SPPB score), and 54% were hypomethylated (i.e., higher methylation level associated with lower SPPB score). Regarding genomic context, approximately half of the identified DMPs were located in open sea regions (51%), while 20% resided within CpG islands. When annotated by gene-centric regions, 17 CpGs (24%) were situated in promoter regions (TSS200/TSS1500), whereas the majority were intergenic (67%). The most significant association was observed at cg20660297, a hypomethylated CpG located within the promoter region of the *KRR1* gene.

**Table 2:** Top 10 skeletal muscle-based DMPs associated with physical function

CpG	Chromosome	Position	Relation to CpG islands	Gene	Gene region	Association with physical function phenotype			
						Beta	SE	*P*	FDR
*SPPB score*									
cg13242895	chr1	216632365	OpenSea	-	-	0.13	0.03	5.54E − 07	0.032
cg18929316	chr17	48099486	Shore	-	-	0.12	0.02	2.13E − 08	0.006
cg02183588	chr22	47549467	OpenSea	-	-	0.1	0.02	1.65E − 06	0.045
cg19808663	chr12	69584871	Shore	CCT2;MIR3913-1	TSS1500;TSS200	0.09	0.02	6.48E − 07	0.032
cg01287200	chr13	26399977	OpenSea	-	-	0.09	0.02	1.28E − 06	0.045
cg20660297	chr12	75512523	Shore	KRR1	TSS1500	− 0.14	0.03	2.58E − 06	0.045
cg25616992	chr8	702342	Shore	-	-	− 0.1	0.02	2.00E − 06	0.045
cg15542016	chr1	204652124	OpenSea	-	-	− 0.1	0.02	2.09E − 08	0.006
cg25326727	chr1	164676015	OpenSea	-	-	− 0.09	0.02	1.12E − 06	0.044
cg11362049	chr11	132101462	OpenSea	-	-	− 0.08	0.02	2.32E − 06	0.045
*6MWT walking speed*									
cg27592988	chr7	128707747	OpenSea	FAM71F1	TSS1500	1.16	0.18	8.94E − 11	7.83E − 05
cg17308762	chr8	41803604	OpenSea	-	-	1.07	0.22	7.41E − 07	0.041
cg25886345	chr4	68311147	OpenSea	YTHDC1	3UTR;exon_17;exon_16	1.03	0.2	2.09E − 07	0.03
cg16037140	chr11	13215319	OpenSea	-	-	1.01	0.18	9.34E − 09	0.003
cg24701190	chr10	118914351	OpenSea	-	-	0.94	0.19	4.12E − 07	0.039
cg07331460	chr7	156116138	OpenSea	-	-	− 0.74	0.15	6.05E − 07	0.039
cg02775617	chr10	31928353	Island	-	-	− 0.65	0.13	6.17E − 07	0.039
cg07696690	chr16	20900256	Island	DCUN1D3;LYRM1	5UTR;exon_1;TSS1500;TSS200	− 0.62	0.12	6.20E − 07	0.039
cg19062112	chr14	102592717	Island	RCOR1	5UTR;exon_1	− 0.54	0.09	7.26E − 09	0.003
*Perceived disability*									
cg02867811	chr6	45327631	OpenSea	RUNX2	TSS1500	1.91	0.35	3.52E − 08	0.008
cg08264579	chr5	90408016	Shore	-	-	1.45	0.28	3.13E − 07	0.045
cg24938788	chr20	6299417	OpenSea	-	-	1.26	0.21	2.01E − 09	0.001
cg13033853	chr9	130939012	Island	FIBCD1	5UTR;exon_1	1.18	0.17	1.39E − 11	1.21E − 05
cg13948486	chr2	162260663	OpenSea	-	-	1.15	0.23	7.47E − 07	0.045
cg14477266	chr12	118052650	OpenSea	-	-	− 0.9	0.18	9.91E − 07	0.048
cg17809941	chr11	74237605	OpenSea	-	-	− 0.72	0.13	2.04E − 08	0.006
cg00369590	chr11	70744064	OpenSea	-	-	− 0.57	0.12	8.19E − 07	0.045
cg22869599	chr7	4235751	OpenSea	-	-	− 0.55	0.11	9.53E − 07	0.048
cg13149730	chr21	46315066	OpenSea	-	-	− 0.49	0.1	7.20E − 07	0.045
*Cardiovascular and lifestyle score*									
cg23530586	chr5	100595733	OpenSea	-	-	0.01	0	9.23E − 09	0.008
cg01817084	chr8	26292049	OpenSea	PPP2R2A	TSS1500	− 0.01	0	1.05E − 07	0.046

The top 10 CpGs for each physical function phenotype are shown (five hypermethylated and five hypomethylated), ranked by beta coefficients

*DMP* differentially methylated position, *SPPB* short physical performance battery, *6MWT* 6-min walk test

A total of 22 significant DMRs were detected. While the mean methylation changes per one-point increase in SPPB score were modest (ranging from −0.01 to + 0.012) (Table 3; Supplemental Table S2), the majority (64%) of these regions were hypermethylated. Genomic annotation revealed that 15 DMRs (68%) overlapped promoter regions (TSS200/TSS1500), indicating that these significant DMRs are concentrated near the transcription start sites (TSS). Notably, *ZBED9*, a gene involved in cell cycle regulation and epithelial cell proliferation, emerged as a key locus in both the DMP (chr6: cg05241721) and DMR (chr6: 28,615,878–28,616,687) analyses. In both instances, lower methylation levels at this locus were significantly associated with superior physical performance (higher SPPB scores).

**Table 3:** Top 10 skeletal muscle-based DMRs associated with physical function

Chromosome	Genomic coordinates	N CpGs	Gene	Gene region	Association with physical function phenotype	
					Mean beta	Combined FDR
*SPPB score*						
chr11	67605481–67605643	3	NDUFV1	TSS1500	0.012	0.008
chr22	45226922–45227185	4	-	-	0.009	2.88E − 04
chr1	44721879–44722274	5	RNU5F-1	TSS1500;exon_1;TSS200	0.008	0.001
chr1	3658140–3658435	3	-	-	0.007	3.20E − 05
chr6	30743784–30744025	10	IER3;FLOT1	3UTR;exon_2;TSS1500	0.006	0.017
chr17	1604349–1605791	11	SLC43A2	exon_1;5UTR;TSS1500;TSS200	− 0.01	0.002
chr22	29313975–29314542	4	RASL10A	exon_2	− 0.007	2.14E − 04
chr5	81301324–81302009	13	CKMT2-AS1;ZCCHC9	exon_1;TSS200;5UTR;TSS1500	− 0.006	0.002
chr1	101847052–101847115	3	OLFM3	TSS200	− 0.005	0.001
chr17	43125677–43126889	14	BRCA1;NBR2	TSS1500;exon_1	− 0.005	0.001
*6MWT walking speed*						
chr5	136079169–136080924	19	VTRNA2-1	TSS200;TSS1500;exon_1	0.126	1.85E − 04
chr1	114099885–114100239	3	-	-	0.114	3.36E − 04
chr18	74498194–74499978	8	CNDP2	exon_1;exon_2;exon_4;5UTR;exon_3;TSS1500;TSS200	0.111	8.61E − 05
chr2	113235117–113235736	8	PAX8-AS1;PAX8	TSS1500;exon_9;TSS200;exon_1	0.109	0.018
chr2	3605056–3605893	6	COLEC11	TSS1500;TSS200	0.105	0.001
chr6	29680384–29682030	22	ZFP57	5UTR;exon_1;TSS200;TSS1500	− 0.146	0.011
chr16	86681393–86681804	3	-	-	− 0.109	0.002
chr5	93573282–93574346	6	-	-	− 0.08	0.009
chr2	118848734–118849819	9	EN1	TSS1500	− 0.072	0.04
chr5	93595107–93596339	8	-	-	− 0.07	0.011
*Handgrip*						
chr18	58862523–58862646	7	ZNF532	TSS1500	− 0.004	2.85E − 06
chr20	37520055–37520869	28	BLCAP;NNAT	5UTR;exon_1;TSS1500;TSS200	− 0.001	1.72E − 04
*Perceived disability*						
chr10	133528366–133528714	3	-	-	− 0.129	0.001

The top 10 DMRs for each physical function phenotype are shown (five hypermethylated and five hypomethylated), ranked by mean beta coefficients

*DMR* differentially methylated regions, *SPPB* short physical performance battery, *6MWT* 6-min walk test

#### Associations with 6MWT walking speed

A total of 16 CpGs were significantly associated with walking speed, with the majority (75%) being hypermethylated (Table 2; Supplemental Table S1; Supplemental Figure S1). Most CpGs were located in open sea regions (69%), and only 25% were located in CpG islands. The majority (62%) were located in intergenic regions, while 19% were located in promoter regions. The top annotated probe was cg27592988, which was a hypermethylated CpG located in *FAM71F1* promoter.

We identified 566 DMRs associated with walking speed (Table 3; Supplemental Table S2). Consistent with the DMP results, these significant DMRs were predominantly hypermethylated (94%). The mean methylation changes ranged from − 0.146 to + 0.126. Gene annotation showed that most DMRs were located within promoter regions (46%), suggesting that a substantial fraction of DMRs may have potential regulatory effects on gene transcription.

A total of three genes were identified in both CpG- and region-level analyses. *TRA2A* (chr7), which encodes a protein involved in RNA splicing regulation, and *MIR1915HG* (chr10), a non-coding RNA gene, were hypermethylated in both DMP and DMR analyses. *RCOR1* (chr14), a transcriptional repressor involved in neuronal and synaptic gene regulation, was hypomethylated in both analyses.

#### Associations with handgrip strength

No individual differentially methylated probes were identified for handgrip strength. However, two DMRs were detected (chr18: 58,862,523–58,862,646 and chr20: 37,520,055–37,520,869), both showing significant hypomethylation associated with greater handgrip strength (Table 3; Supplemental Table S2; Supplemental Figure S2). These regions contained a relatively high number of potentially co-methylated probes (7 and 28 probes, respectively). However, the mean methylation changes were small, ranging from − 0.004 to − 0.001. Both DMRs overlapped promoter regions (TSS1500 and TSS200), suggesting a modest but significant potential role in gene transcription.

#### Associations with perceived disability

A total of 19 CpGs were significantly associated with the PAT-D score, with slightly more hypomethylated probes than hypermethylated probes (58% vs. 42%). Most CpGs (68%) were located in open sea regions, followed by CpG islands (16%) and shore (16%). With respect to gene transcript regions, 16 CpGs (84%) were mapped to intergenic regions. The strongest association was observed for hypermethylated cg02867811, located in the promoter region of *RUNX2*. (Table 2; Supplemental Table S1; Supplemental Figure S3).

Only one hypomethylated DMR was significantly associated with the PAT-D score; however, no genes were annotated to this region. (Table 3; Supplemental Table S2).

#### Associations with cardiovascular and lifestyle score

We identified one hypermethylated and one hypomethylated CpG associated with the modified LE8 score. Both CpGs were located in open sea regions. The hypomethylated CpG cg01817084 mapped to the promoter of *PPP2R2A*. No significant DMRs were identified (Table 2; Supplemental Table S1; Supplemental Figure S4).

##### Key DMPs/DMRs

Supplemental Table S3 summarizes key DMPs/DMRs associated with physical function, their overlap with published GWAS findings, and the direction of DNA methylation associations for the highlighted genes.

##### Genomic inflation

Following BACON correction, genomic inflation factor was modest across all analyses (*λ* = 1.01—1.14), indicating no evidence of meaningful inflation or deflation of the test statistics. Q-Q plots and corresponding *λ* values are shown in Supplemental Figure S5.

### Enrichment analysis

**Fig. 3:**
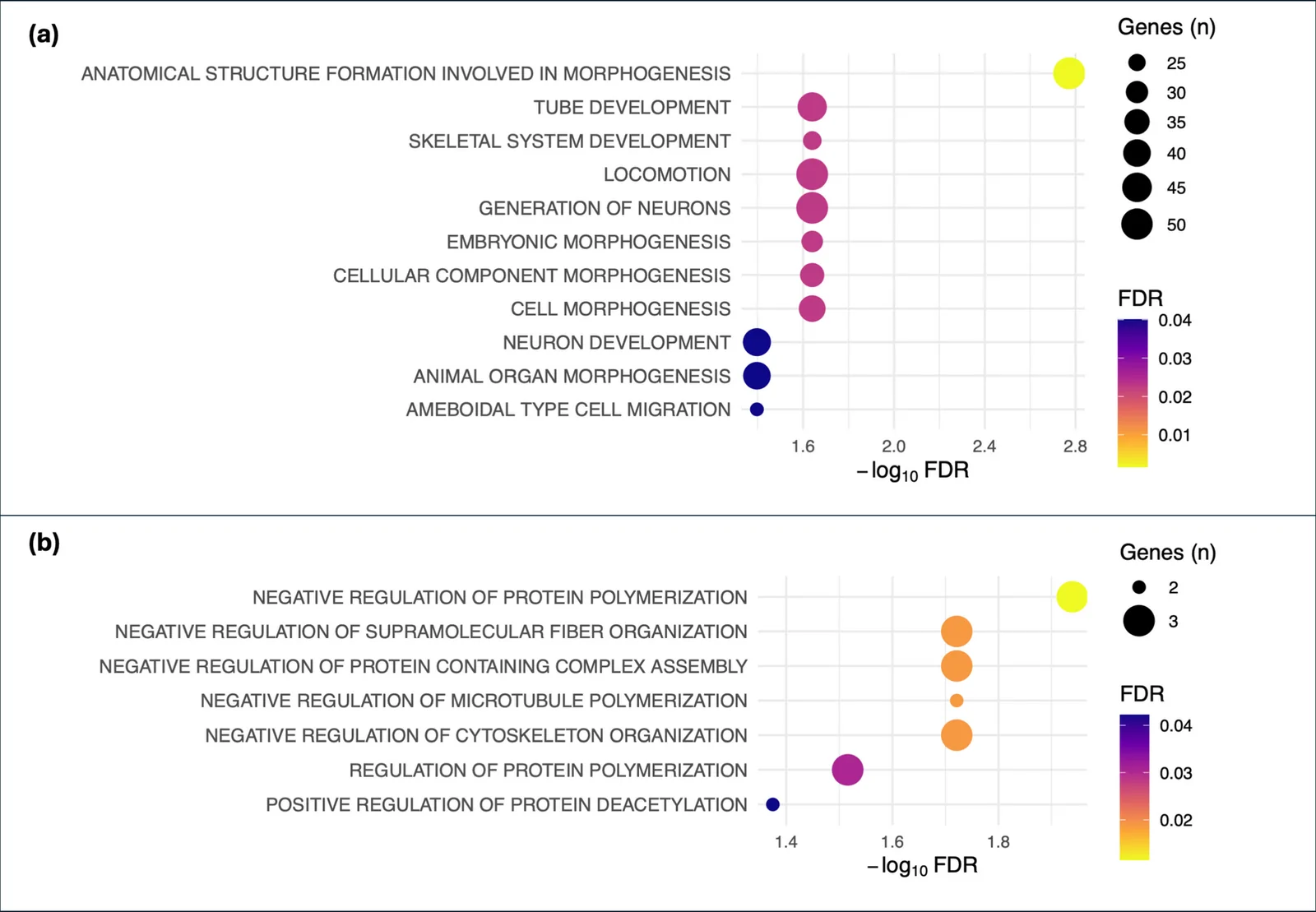
Gene set enrichment analysis of differentially methylated positions (DMPs) and regions (DMRs) associated with **a** 6MWT walking speed and **b** handgrip strength. Gene sets were derived from the biological process category of gene ontology in MSigDB. Dot size represents the number of overlapping genes per gene ontology term, color indicates FDR-adjusted significance. No significant enrichment was identified for other physical function phenotypes

Gene-set enrichment analyses were stratified by methylation direction for both DMPs and DMRs. Suggestive hypermethylated DMRs associated with 6MWT walking speed were enriched for 11 GO terms primarily centered on neuro-musculoskeletal development. These included anatomical structure formation involved in morphogenesis, cell and cellular component morphogenesis, locomotion, neuron generation and development, and skeletal system development (Fig. 3). Conversely, hypomethylated CpGs associated with handgrip strength were significantly enriched in seven GO biological processes, primarily related to cytoskeletal dynamics and protein assembly. Key pathways included the regulation of protein polymerization and deacetylation, protein-containing complex assembly, cytoskeleton organization, and supramolecular fiber organization. No significant GO or KEGG enrichment was observed for gene sets associated with the remaining physical function phenotypes.

## Discussion

Aging is a driver of functional decline and disability with marked variability in the rate and severity of physical performance decline among older adults [27]. While some individuals maintain high levels of mobility well into their eighth or ninth decade, others experience rapid decline [6], yet the biological mechanisms underlying this heterogeneity remain largely unexplored. Our analysis demonstrates that altered DNA methylation in skeletal muscle may underlie this observed variability, independent of known risk factors. Notably, walking speed, a robust indicator of overall health and mortality, was positively associated with multiple methylated regions. A substantial proportion of these signals mapped to promoter regions, suggesting that the muscle epigenome plays a pivotal regulatory role in gene transcription related to physical performance. Specifically, we identified significant associations of genes critical to musculoskeletal health, such as *RUNX2 *[28] and *FOXL1* [29, 30] (bone density), *IGFBP3* [31, 32] (muscle mass), and *NEK1* [33] and *SHANK1* [34] (neurological function). Gene-set enrichment analyses indicated biological processes contributing to protein assembly, cytoskeleton organization, tissue formation, and skeletal and neuronal development. Collectively, these muscle-specific methylation signatures may provide a molecular framework for understanding inter-individual differences in physical performance and point to pathways that could be targeted to preserve independence and physical fitness in older adults.

The loss of independence and functional decline in older adults are intrinsically related to skeletal muscle health; however, research on human skeletal muscle remains limited, largely due to the difficulty of obtaining sufficient numbers and quantities of muscle tissue from population-based studies. To date, EWAS studies investigating physical function have relied almost exclusively on whole blood. For instance, Marioni et al. analyzed 450,726 CpGs in 1091 older adults (mean age 70) but found no significant CpGs linked to grip strength or walking speed [8]. Similarly, Bell et al. profiled 26,690 CpG sites in 172 female twins (aged 32–80) and reported no significant associations with grip strength [11]. Our study suggests that these null findings were likely a consequence of tissue specificity. By examining DNA methylation in skeletal muscle directly, we identified multiple differentially methylated CpGs and regions associated with SPPB score and 6MWT walking speed. A few significant hits were associated with grip strength, perceived disability, and lifestyle health. While most significant DMPs were located in open sea regions and intergenic areas, a significant proportion of DMRs overlapped with promoters, suggesting that coordinated epigenetic remodeling in muscle may directly regulate the transcription of genes essential for locomotion.

Previous GWAS have demonstrated that genetic factors contribute substantially to physical function, accounting for approximately 50% of the variability in handgrip strength [35] and 17–20% of the variance in walking speed and endurance [36, 37]. Interestingly, several loci (e.g., *RUNX2*, *FOXL1* and *IGSF23*) were confirmed in our study, suggesting that these genes may be epigenetically modulated to influence physical function. For instance, *RUNX2* [28], a gene associated with bone mineral density in previous GWAS, was our top hit for perceived disability, exhibiting significant hypermethylation at cg02867811. Other bone-related loci, including *FOXL1* [29, 30] and *ESR1* [38] (candidate genes associated with bone mineral density or osteoporosis in previous GWAS) as well as *IGSF23* [39] (gene involved in bone remodeling and skeletal homeostasis), were identified as significant differentially methylated regions (DMRs) associated with walking speed in our study. Furthermore, *IGFBP3* [31, 32], a candidate gene for muscle mass, mapped to a hypermethylated DMR (chr7, 45921235–45922361) significantly associated with walking speed. Additionally, individual CpGs pinpointed novel pathways: cg20660297(*KRR1),* involved in ribosome assembly [40], and cg01817084 (*PPP2R2A*), linked to cell growth and DNA damage response [41, 42], were both hypomethylated in relation to SPPB and lifestyle health scores. These findings suggest that the identified genes may serve as important epigenetic interfaces through which environmental or lifestyle factors modulate gene expression to influence physical fitness in older adults alongside genetic contributions.

The observed larger number of DMRs identified for the 6MWT walking speed compared with the other physical performance phenotypes likely reflects both biological and methodological factors. Biologically, the 6MWT is a highly integrative measure of functional capacity, capturing the coordinated output of multiple physiological systems, including cardiovascular, neuromuscular, respiratory, and metabolic functions. Thus, it may be underpinned by broader epigenetic alterations than phenotypes that reflect more circumscribed aspects of physical function, such as grip strength or lower-extremity function as measured by SPPB score. This systemic biological complexity may facilitate the detection of widespread regional methylation changes (i.e., DMRs) in skeletal muscle. Methodologically, the 6MWT is a continuous measure, which may provide greater statistical power to detect methylation associations than ordinal or score-based measures with discrete or ceiling values. Taken together, while we cannot fully disentangle the biological from the statistical contributions, the biological coherence and functional relevance of the implicated genomic regions support the interpretation that the identified DMRs represent meaningful epigenetic signals rather than statistical noise or confounding influences.

Our GO analysis revealed that differentially methylated genes associated with walking speed are implicated in biological processes related to cell and tissue morphogenesis, organ and skeletal system development, and locomotion. Notably, pathways related to neuron generation and development were significantly enriched. We identified *RCOR1*, a transcriptional repressor involved in neuronal and synaptic gene regulation [43], as being hypomethylated in relation to walking speed in both our DMP and DMR analyses; in addition, *NEK1* (implicated in amyotrophic lateral sclerosis, neurodegeneration, and DNA damage response [33]) and *SHANK1* (implicated in neurodevelopment and synaptic function [34]) hypomethylation was both associated with better physical function (SPPB score). This is consistent with previous GWAS findings that implicate synaptic function and neuronal development as crucial biological pathways underlying gait speed [44]. These results further corroborate with previous research demonstrating that nervous system genes significantly influence handgrip strength [45], underscoring the vital neural contribution to muscle function. Our enrichment analysis of handgrip strength–associated CpGs highlighted biological processes involved in protein and cytoskeletal dynamics. This evidence is in line with previous genetic studies linking protein catabolism to handgrip strength [45]. Together, these findings suggest that the muscle methylome regulates neuronal signaling and protein turnover, both of which are essential for preserving physical function in older adults.

Our study has several key strengths. Most notably, we quantified DNA methylation using the Illumina EPIC v2 array, providing high-resolution coverage of over 870,000 CpG sites within human skeletal muscle, the primary tissue driving mobility and strength. This offers more direct biological insights into physical function than blood-based methylation analysis. Furthermore, our cohort consists of adults free from major chronic diseases, such as cardiovascular disease, diabetes, and cancer, thereby minimizing biological noise from comorbidities that can confound epigenetic signatures. Finally, we assessed a comprehensive battery of objective physical performance measures, including the SPPB, grip strength, and 6MWT walking endurance, ensuring a robust evaluation of both upper and lower body functional capacity.

Several limitations should also be acknowledged. First, the relatively modest sample size represents an important limitation of the present study. Restricted statistical power increases the likelihood of both false-positive and false-negative findings. The reproducibility and generalizability of our findings across different populations, age groups, and tissue sources therefore warrant investigation in future larger-scale studies. It should be noted, however, that our sample size is consistent with, and in several cases exceeds, those reported in comparable skeletal muscle epigenomic studies, reflecting the practical constraints imposed by the invasive nature of muscle biopsy collection. Second, as nicotine exposure and lipid components of the LE8 were unavailable, our modified score may not be fully comparable to standard metric. However, given that the six available components capture the majority of variability in cardiovascular and lifestyle health, and the generally healthy status of the participants, we do not anticipate that the absence of these two components substantially affected our results. Third, while excluding individuals with major comorbidities reduced confounding, it may also introduce a healthy volunteer bias, potentially limiting the generalizability of our findings. Fourth, we lacked complete data on key behavioral factors, such as smoking history and dietary patterns, and we cannot exclude the possibility of residual confounding from these unmeasured behaviors. Fifth, our findings were derived from a single cohort without external validation, which may limit generalizability and clinical translation. However, large skeletal muscle epigenetic datasets remain scarce due to the invasive nature of tissue acquisition. In the absence of an immediately available replication dataset, we propose the following validation framework: (1) Collaborative data-sharing initiatives and meta-analysis across existing muscle biopsy cohorts would provide the most direct opportunity for replication; (2) cross-tissue validation using blood-based methylation data from large cohorts could help determine whether identified signals have tissue-independent relevance to physical function, while acknowledging inherent tissue-specificity constraints; and (3) prospective studies with repeated epigenetic and functional assessments will be essential for evaluating the predictive validity of the identified DMRs as early markers functional decline. Sixth, we used SVA to account for potential muscle fiber-type heterogeneity; however, SVA is designed to capture latent sources of unwanted variation and may only explain a proportion of this variability, and thus, residual confounding cannot be entirely ruled out. Lastly, the cross-sectional design precludes any causal inference; longitudinal studies are required to establish the temporal relationship between skeletal muscle methylation and functional decline.

## Conclusions

In summary, we identified distinct DNA methylation signatures in human skeletal muscle that were significantly associated with physical function in healthy older adults. Our enrichment analysis revealed multiple biological processes, ranging from protein assembly and cytoskeleton organization, skeletal muscle tissue organization, to neuroskeletal development, offering a molecular landscape for the muscle aging process. These findings highlight specific epigenetic pathways that may serve as targets for pharmacological or behavioral interventions for mitigating functional decline and promoting healthy aging. While these results are promising, validating these findings in larger, more diverse populations is essential for translating these muscle-specific epigenetic signatures into clinical practice.

## Supplementary information

Below is the link to the electronic supplementary material.

ESM 1 (DOCX 1.54 MB)

## Data Availability

The data that support the findings of this study are available from the corresponding author upon reasonable request.

## References

[1] Jones CH, Dolsten M. Healthcare on the brink: navigating the challenges of an aging society in the United States. NPJ Aging Mech Dis. 2024;10:22. 10.1038/s41514-024-00148-2. PMC10998868 38582901

[2] Cawthon PM, Travison TG, Manini TM, Patel S, Pencina KM, Fielding RA, et al. Establishing the link between lean mass and grip strength cut points with mobility disability and other health outcomes: proceedings of the sarcopenia definition and outcomes consortium conference. J Gerontol A Biol Sci Med Sci. 2020;75:1317–23. 10.1093/gerona/glz081. PMC7447857 30869772

[3] Manini TM, Buford TW, Kairalla JA, McDermott MM, Vaz Fragoso CA, Fielding RA, et al. Meta-analysis identifies mitochondrial DNA sequence variants associated with walking speed. Geroscience. 2018;40:497–511. 10.1007/s11357-018-0043-x. PMC6294723 30338417

[4] Callahan KE, Lovato L, Miller ME, Marsh AP, Fielding RA, Gill TM, et al. Self-reported physical function as a predictor of hospitalization in the lifestyle interventions and independence for elders study. J Am Geriatr Soc. 2018;66:1927–33. 10.1111/jgs.15468. PMC6277208 30281796

[5] Marsh AP, Applegate WB, Guralnik JM, Jack Rejeski W, Church TS, Fielding RA, et al. Hospitalizations during a physical activity intervention in older adults at risk of mobility disability: analyses from the lifestyle interventions and independence for elders randomized clinical trial. J Am Geriatr Soc. 2016;64:933–43. 10.1111/jgs.14114. PMC4887151 27225353

[6] Rothermund K, Englert C, Gerstorf D. Explaining variation in individual aging, its sources, and consequences: a comprehensive conceptual model of human aging. Gerontology. 2023;69:1437–47. 10.1159/000534324. PMC10711769 37769642

[7] Jones MJ, Goodman SJ, Kobor MS. DNA methylation and healthy human aging. Aging Cell. 2015;14:924–32. 10.1111/acel.12349. PMC4693469 25913071

[8] Marioni RE, Shah S, McRae AF, Ritchie SJ, Muniz-Terrera G, Harris SE, et al. The epigenetic clock is correlated with physical and cognitive fitness in the Lothian Birth Cohort 1936. Int J Epidemiol. 1936;44:1388–96. 10.1093/ije/dyu277. PMC4588858 25617346

[9] Föhr T, Törmäkangas T, Lankila H, Viljanen A, Rantanen T, Ollikainen M, et al. The association between epigenetic clocks and physical functioning in older women: a 3-year follow-up. J Gerontol A Biol Sci Med Sci. 2022;77:1569–76. 10.1093/gerona/glab270. PMC9373966 34543398

[10] Collerton J, Gautrey HE, van Otterdijk SD, Davies K, Martin-Ruiz C, von Zglinicki T, et al. Acquisition of aberrant DNA methylation is associated with frailty in the very old: findings from the Newcastle 85+ Study. Biogerontology. 2014;15:317–28. 10.1007/s10522-014-9500-9. 24770842

[11] Bell JT, Tsai PC, Yang TP, Pidsley R, Nisbet J, Glass D, et al. Epigenome-wide scans identify differentially methylated regions for age and age-related phenotypes in a healthy ageing population. PLoS Genet. 2012;8:e1002629. 10.1371/journal.pgen.1002629. PMC3330116 22532803

[12] Zykovich A, Hubbard A, Flynn JM, Tarnopolsky M, Fraga MF, Kerksick C, et al. Genome-wide DNA methylation changes with age in disease-free human skeletal muscle. Aging Cell. 2014;13:360–6. 10.1111/acel.12180. PMC3954952 24304487

[13] Guralnik JM, Simonsick EM, Ferrucci L, Glynn RJ, Berkman LF, Blazer DG, et al. A short physical performance battery assessing lower extremity function: association with self-reported disability and prediction of mortality and nursing home admission. J Gerontol. 1994;49:M85-94. 10.1093/geronj/49.2.m85. 8126356

[14] Laboratories ACoPSfCPF. ATS statement: guidelines for the six-minute walk test. Am J Respir Crit Care Med. 2002;166:111–7. 10.1164/ajrccm.166.1.at1102. 12091180

[15] Otadi K, Malmir K. Normative reference values for the six-minute walk test in older adults: a systematic review and meta-analysis. Arch Gerontol Geriatr. 2026;140:106026. 10.1016/j.archger.2025.106026. 41027267

[16] Porto JM, Nakaishi APM, Cangussu-Oliveira LM, Freire Júnior RC, Spilla SB, Abreu DCC. Relationship between grip strength and global muscle strength in community-dwelling older people. Arch Gerontol Geriatr. 2019;82:273–8. 10.1016/j.archger.2019.03.005. 30889410

[17] Wang T, Wu Y, Li W, Li S, Sun Y, Li S, et al. Weak grip strength and cognition predict functional limitation in older Europeans. J Am Geriatr Soc. 2019;67:93–9. 10.1111/jgs.15611. 30357802

[18] Rejeski WJ, Ip EH, Marsh AP, Miller ME, Farmer DF. Measuring disability in older adults: the International Classification System of Functioning, Disability and Health (ICF) framework. Geriatr Gerontol Int. 2008;8:48–54. 10.1111/j.1447-0594.2008.00446.x. PMC5118869 18713189

[19] Aryee MJ, Jaffe AE, Corrada-Bravo H, Ladd-Acosta C, Feinberg AP, Hansen KD, et al. Minfi: a flexible and comprehensive Bioconductor package for the analysis of Infinium DNA methylation microarrays. Bioinformatics. 2014;30:1363–9. 10.1093/bioinformatics/btu049. PMC4016708 24478339

[20] Pidsley R, Y Wong CC, Volta M, Lunnon K, Mill J, Schalkwyk LC. A data-driven approach to preprocessing Illumina 450K methylation array data. BMC Genomics. 2013;14:293. 10.1186/1471-2164-14-293. PMC3769145 23631413

[21] Leek JT, Johnson WE, Parker HS, Jaffe AE, Storey JD. The sva package for removing batch effects and other unwanted variation in high-throughput experiments. Bioinformatics. 2012;28:882–3. 10.1093/bioinformatics/bts034. PMC3307112 22257669

[22] Ritchie ME, Phipson B, Wu D, Hu Y, Law CW, Shi W, et al. limma powers differential expression analyses for RNA-sequencing and microarray studies. Nucleic Acids Res. 2015;43:e47. 10.1093/nar/gkv007. PMC4402510 25605792

[23] van Iterson M, van Zwet EW, Heijmans BT. Controlling bias and inflation in epigenome- and transcriptome-wide association studies using the empirical null distribution. Genome Biol. 2017;18:19. 10.1186/s13059-016-1131-9. PMC5273857 28129774

[24] Peters TJ, Buckley MJ, Statham AL, Pidsley R, Samaras K, V Lord R, et al. De novo identification of differentially methylated regions in the human genome. Epigenetics Chromatin. 2015;8:6. 10.1186/1756-8935-8-6. PMC4429355 25972926

[25] Lawrence M, Huber W, Pagès H, Aboyoun P, Carlson M, Gentleman R, et al. Software for computing and annotating genomic ranges. PLoS Comput Biol. 2013;9:e1003118. 10.1371/journal.pcbi.1003118. PMC3738458 23950696

[26] Watanabe K, Taskesen E, van Bochoven A, Posthuma D. Functional mapping and annotation of genetic associations with FUMA. Nat Commun. 2017;8:1826. 10.1038/s41467-017-01261-5. PMC5705698 29184056

[27] Newman AB. The epidemiology and societal impact of aging-related functional limitations: a looming public health crisis. J Gerontol A Biol Sci Med Sci. 2023;78:4–7. 10.1093/gerona/glad021. PMC10272977 37325965

[28] Estrada K, Styrkarsdottir U, Evangelou E, Hsu YH, Duncan EL, Ntzani EE, et al. Genome-wide meta-analysis identifies 56 bone mineral density loci and reveals 14 loci associated with risk of fracture. Nat Genet. 2012;44:491–501. 10.1038/ng.2249. PMC3338864 22504420

[29] Rivadeneira F, Styrkársdottir U, Estrada K, Halldórsson BV, Hsu YH, Richards JB, et al. Twenty bone-mineral-density loci identified by large-scale meta-analysis of genome-wide association studies. Nat Genet. 2009;41:1199–206. 10.1038/ng.446. PMC2783489 19801982

[30] Kim BJ, Ahn SH, Kim HM, Ikegawa S, Yang TL, Guo Y, et al. Replication of Caucasian loci associated with osteoporosis-related traits in East Asians. J Bone Metab. 2016;23:233–42. 10.11005/jbm.2016.23.4.233. PMC5153380 27965945

[31] Yang CW, Li TC, Li CI, Liu CS, Lin CH, Lin WY, et al. Insulinlike growth factor-1 and its binding protein-3 polymorphisms predict circulating IGF-1 level and appendicular skeletal muscle mass in Chinese elderly. J Am Med Dir Assoc. 2015;16:365–70. 10.1016/j.jamda.2014.11.015. 25548030

[32] Pratt J, Boreham C, Ennis S, Ryan AW, De Vito G. Genetic associations with aging muscle: a systematic review. Cells. 2019. 10.3390/cells9010012. PMC7016601 31861518

[33] Kenna KP, van Doormaal PT, Dekker AM, Ticozzi N, Kenna BJ, Diekstra FP, et al. NEK1 variants confer susceptibility to amyotrophic lateral sclerosis. Nat Genet. 2016;48:1037–42. 10.1038/ng.3626. PMC5560030 27455347

[34] Monteiro P, Feng G. SHANK proteins: roles at the synapse and in autism spectrum disorder. Nat Rev Neurosci. 2017;18:147–57. 10.1038/nrn.2016.183. 28179641

[35] Zempo H, Miyamoto-Mikami E, Kikuchi N, Fuku N, Miyachi M, Murakami H. Heritability estimates of muscle strength-related phenotypes: a systematic review and meta-analysis. Scand J Med Sci Sports. 2017;27:1537–46. 10.1111/sms.12804. 27882617

[36] Pajala S, Era P, Koskenvuo M, Kaprio J, Alén M, Tolvanen A, et al. Contribution of genetic and environmental factors to individual differences in maximal walking speed with and without second task in older women. J Gerontol A Biol Sci Med Sci. 2005;60:1299–303. 10.1093/gerona/60.10.1299. 16282563

[37] Ortega-Alonso A, Pedersen NL, Kujala UM, Sipilä S, Törmäkangas T, Kaprio J, et al. A twin study on the heritability of walking ability among older women. J Gerontol A Biol Sci Med Sci. 2006;61:1082–5. 10.1093/gerona/61.10.1082. 17077203

[38] Koller DL, Zheng HF, Karasik D, Yerges-Armstrong L, Liu CT, McGuigan F, et al. Meta-analysis of genome-wide studies identifies WNT16 and ESR1 SNPs associated with bone mineral density in premenopausal women. J Bone Miner Res. 2013;28:547–58. 10.1002/jbmr.1796. PMC3691010 23074152

[39] Yuan Y, Yang L, Liu T, Zhang H, Lu Q. Osteoclastogenesis inhibition by mutated IGSF23 results in human osteopetrosis. Cell Prolif. 2019;52:e12693. 10.1111/cpr.12693. PMC6869366 31560140

[40] Gromadka R, Rytka J. The KRR1 gene encodes a protein required for 18S rRNA synthesis and 40S ribosomal subunit assembly in *Saccharomyces cerevisiae*. Acta Biochim Pol. 2000;47:993–1005. 11996121

[41] Kalev P, Simicek M, Vazquez I, Munck S, Chen L, Soin T, et al. Loss of PPP2R2A inhibits homologous recombination DNA repair and predicts tumor sensitivity to PARP inhibition. Cancer Res. 2012;72:6414–24. 10.1158/0008-5472.CAN-12-1667. 23087057

[42] Panicker N, Coutman M, Lawlor-O’Neill C, Kahl RGS, Roselli S, Verrills NM. Knockout mice reveal that protein phosphatase 2A regulatory subunit, PP2A-B55α, is an essential regulator of neuronal and epidermal embryonic development. Front Cell Dev Biol. 2020;8:358. 10.3389/fcell.2020.00358. PMC7290052 32582689

[43] Monaghan CE, Nechiporuk T, Jeng S, McWeeney SK, Wang J, Rosenfeld MG, et al. REST corepressors RCOR1 and RCOR2 and the repressor INSM1 regulate the proliferation-differentiation balance in the developing brain. Proc Natl Acad Sci U S A. 2017;114:E406–15. 10.1073/pnas.1620230114. PMC5255626 28049845

[44] Ben-Avraham D, Karasik D, Verghese J, Lunetta KL, Smith JA, Eicher JD, et al. The complex genetics of gait speed: genome-wide meta-analysis approach. Aging (Albany NY). 2017;9:209–46. 10.18632/aging.101151. PMC5310665 28077804

[45] Willems SM, Wright DJ, Day FR, Trajanoska K, Joshi PK, Morris JA, et al. Large-scale GWAS identifies multiple loci for hand grip strength providing biological insights into muscular fitness. Nat Commun. 2017;8:16015. 10.1038/ncomms16015. PMC5510175 29313844

