# Supplementary material for: DNA methylation signatures in skeletal muscle associated with physical function in healthy older adults
Source: GeroScience. 2026 Aug 8;48(6):7597–612. doi: 10.1007/s11357-026-02410-9 (PMC13638902; doi:10.1007/s11357-026-02410-9)
Supplement: Supplementary file 1 [file 11357_2026_2410_MOESM1_ESM.docx]

**Supplemental Materials**

**Supplemental Methods**

1. Study population - eligibility criteria for enrollment

2. Protocol for purification of genomic DNA from human skeletal muscle

3. Preprocessing and quality control procedures for DNA methylation data

4. Modified Life’s Essential 8 scoring procedures

**Supplemental Figure**

1. Supplemental Figure S1. Differentially methylated positions (DMPs) and regions (DMRs) in human skeletal muscle associated with walking speed

2. Supplemental Figure S2. Differentially methylated positions (DMPs) and regions (DMRs) in human skeletal muscle associated with handgrip strength

3. Supplemental Figure S3. Differentially methylated positions (DMPs) and regions (DMRs) in human skeletal muscle associated with perceived disability

4. Supplemental Figure S4. Differentially methylated positions (DMPs) and regions (DMRs) in human skeletal muscle associated with cardiovascular and lifestyle health

5. Supplemental Figure S5. Quantile–quantile (Q–Q) plots and genomic inflation factors (λ) after genomic correction

**Supplemental Tables**

1. Supplemental Table S1. Skeletal muscle-based DMPs associated with physical function

2. Supplemental Table S2. Skeletal muscle-based DMRs associated with physical function

3. Supplemental Table S3. Key DMPs/DMRs and their associations with physical function phenotype

**References**

**Supplemental Methods**

**1. Study Population - Eligibility Criteria for Enrollment**

This study enrolled 114 participants aged 70 to 80 years and entered the follow-up stage. In the current study, after excluding 17 participants without muscle biopsy sample and 5 with missing data on physical function phenotypes, a total of 92 participants were included in the data analysis.

**Inclusion criteria**

- 70-80 years old
- Short Physical Performance Battery (SPPB) score ≤ 9 (Low Functioning Group) or 11-12 (High Functioning Group)
- Physically inactive lifestyle (i.e., <150 min/week of moderate-intensity aerobic exercise) and no active participation in a formal exercise program within the past 3 months (i.e., brisk walks are considered formal exercise, but leisurely walks are not)
- Ability to walk one block (400 meters)
- Mini-Mental State Examination (MMSE) score ≥ 24
- Willingness to undergo all testing procedures

**Exclusion criteria**

- Failure to provide informed consent
- Use of walking assistance devices such as cane, crutches, walkers
- Active treatment for cancer, stroke (< 6 months), peripheral vascular disease, coronary artery disease, myocardial infarction (< 6 months), congestive heart failure (stage III or IV), valvular heart disease, COPD
- Allergy to lidocaine
- SPPB score of 10
- Major psychiatric disease
- Malabsorption (e.g., celiac disease)
- Major gastrointestinal disorders (e.g., peptic ulcer, chronic gastritis/duodenitis, inflammatory bowel disease)
- Chronic inflammatory disorders (e.g., autoimmune disease, chronic infections)
- Splenectomy
- Hemolytic anemia, severe anemia, liver or renal disease within the past 3 years
- Diabetes (self-report) or fasting glucose > 126 mg/dL
- Severe osteoarthritis, fracture in upper or lower extremity (< 6 months), upper or lower extremity amputation
- Neuromuscular disorders including ALS, Parkinson’s disease, muscular dystrophy, multiple sclerosis
- Excessive alcohol use (i.e., > 14 drinks/week) or alcohol abuse (i.e., > 5 drinks/day for males or > 4 drinks/day for females)
- Other substance abuse within the past 3 years
- Smoking history in the past 3 years
- Current use of anabolic hormones (i.e., growth hormones or testosterone) or anticoagulants
- Unwilling or study physician’s advice not to stop antiplatelet drug 5 to 7 days before and 2 to 3 days after the muscle biopsy
- Participating in another clinical trial or has received an investigational product within 30 days prior to screening/enrollment

**Temporary Exclusion criteria**

- Recent bacterial/viral infection (< 3 weeks)
- Acute febrile illness in the past 2 months
- Resting heart rate > 120 bpm
- High blood pressure (i.e., ≥ 180/100 mmHg) at screening visit
- Major surgery or hip/knee replacement (< 6 months)

**2. Protocol for purification of genomic DNA from human skeletal muscle**

(QIAGEN: DNeasy Blood & Tissue Kit Spin-Column. REF: 69504)

Preparation:

1. Buffer ATL and Buffer AL may form precipitates upon storage. If necessary, warm to 56°C until the precipitates have fully dissolved.
2. Buffer AW1 and Buffer AW2 are supplied as concentrates. Before using for the first time, add the appropriate amount of ethanol (96–100%) as indicated on the bottle to obtain a working solution.
3. Preheat a thermomixer, shaking water bath or rocking platform to 56°C for use in step 2. If using frozen tissue, equilibrate the sample to room temperature (15–25°C).
4. Avoid repeated thawing and freezing of samples, because this will lead to reduced DNA size.

Procedure

1. Cut about 25 mg tissue into small pieces, and place in a 1.5 mL Eppendorf safe-lock tube. Add 180 µl of Buffer ATL.
2. Homogenize samples use “NEXT ADVANCE Bullet Blender homogenizer” in 180uL ATL

Use 1.5 mL Eppendorf safe-lock tubes and stainless-steel beads (0.5~2mm)

i. About ½ of the little white scoop is sufficient (about 20 beads)
ii. On Setting 8, homogenize samples in (30sec) bouts. Remove and place on ice bucket for 5 minutes between homogenization step. (1 or 2 rounds of homogenization is often sufficient)

3. Add 20 µl Proteinase K. Mix thoroughly by vortexing, place in a thermomixer and incubate at 56°C until the tissue is completely lysed. Lysis time varies depending on the type of tissue processed. Lysis is usually complete in 1–3 h or. If it is more convenient, samples can be lysed overnight; this will not affect them adversely.

**Optional**: If RNA-free genomic DNA is required, add 4 µl RNase A (100 mg/ml), mix by vortexing, and incubate for 2 min at room temperature (15–25°C) before continuing with step 3.

4. Vortex for 15 s. Add 200 µl Buffer AL to the sample, and mix thoroughly by vortexing. Then add 200 µl ethanol (96–100%), and mix again thoroughly by vortexing. It is essential that the sample, Buffer AL, and ethanol are mixed immediately and thoroughly by vortexing or pipetting to yield a homogeneous solution. Buffer AL and ethanol can be premixed and added together in one step to save time when processing multiple samples. A white precipitate may form on addition of Buffer AL and ethanol. This precipitate does not interfere with the DNeasy procedure. Some tissue types (e.g., spleen, lung) may form a gelatinous lysate after addition of Buffer AL and ethanol. In this case, vigorously shaking or vortexing the preparation is recommended.
5. Pipet the mixture from step 3 (including any precipitate) into the DNeasy Mini spin column placed in a 2 ml collection tube (provided). Centrifuge at ≥6000 x g (8000 rpm) for 1 min. Discard flow-through and collection tube. *
6. Place the DNeasy Mini spin column in a new 2 ml collection tube (provided), add 500 µl Buffer AW1, and centrifuge for 1 min at ≥6000 x g (8000 rpm). Discard flow-through and collection tube. *
7. Place the DNeasy Mini spin column in a new 2 ml collection tube (provided), add 500 µl Buffer AW2, and centrifuge for 3 min at 20,000 x g (14,000 rpm) to dry the DNeasy membrane. Discard flow-through and collection tube. It is important to dry the membrane of the DNeasy Mini spin column, since residual ethanol may interfere with subsequent reactions. This centrifugation step ensures that no residual ethanol will be carried over during the following elution. Following the centrifugation step, remove the DNeasy Mini spin column carefully so that the column does not come into contact with the flow-through, since this will result in carryover of ethanol. If carryover of ethanol occurs, empty the collection tube, then reuse it in another centrifugation for 1 min at 20,000 x g (14,000rpm).
8. Place the DNeasy Mini spin column in a clean 1.5 ml or 2 ml microcentrifuge tube (not provided), and pipet 200 µl Buffer AE directly onto the DNeasy membrane. Incubate at room temperature for 1 min, and then centrifuge for 1 min at ≥ 6000 x g (8000 rpm) to elute. Elution with 100 µl (instead of 200 µl) increases the final DNA concentration in the eluate, but also decreases the overall DNA yield
9. Recommended: For maximum DNA yield, repeat elution once as described in step 7. This step leads to increased overall DNA yield. A new microcentrifuge tube can be used for the second elution step to prevent dilution of the first eluate. Alternatively, to combine the eluates, the microcentrifuge tube from step 7 can be reused for the second elution step. Note: Do not elute more than 200 µl into a 1.5 ml microcentrifuge tube because the DNeasy Mini spin column will come into contact with the eluate.

**3. Preprocessing and quality control procedures for DNA methylation data**

Raw data were processed using the R packages *minfi^1^* (v.1.50.0) and *wateRmelon^2^* (v2.10.0). Samples were excluded if: 1) proportion of loci detection rate <95%, 2) mean intensity of methylated or unmethylated signals were three standard deviations above or below the mean, 3) bisulfite conversion efficiency <80%, 4) there was a mismatch between reported and predicted sex, or 5) detection *P*>0.05 in more than 1% of probes. Probes were excluded if: 1) 1% of samples with a detection *P*>0.05, 2) bead count <3 in 5% of the samples, 3) probes are on sex chromosomes, 4) probe sequences contain SNPs with minor allele frequency (MAF)≥0.05, or 5) they belong to previously reported cross-reactive probes. For probes mapping to the same genomic site, we retained the CpG with the highest quality (based on detection *p*-values and bead counts). Non-CpG sites were excluded from further analysis. The subsequent normalization of DNA methylation data was first performed using quantile normalization method implemented in the R package *wateRmelon*. Then we applied the beta-mixture quantile normalization (BMIQ) approach implemented in the R package *wateRmelon* to normalize beta values of type I and type II design probes within the Illumina arrays. Given that methylation M values (logit transformation of methylation beta values) have better statistical properties (i.e., homoscedasticity) for linear regression models, we derived M values for the downstream analysis.

**4. Modified Life’s Essential 8 Scoring Procedures**

| **Domain** | **Metric** | **Category / Definition** | **Points** |
| --- | --- | --- | --- |
| Health Factors | Blood Pressure | - SBP <120 mmHg and DBP <80 mmHg | 100 |
|  |  | - SBP 120–129 mmHg and DBP <80 mmHg | 75 |
|  |  | - SBP 130–139 mmHg or DBP 80–89 mmHg | 50 |
|  |  | - SBP 140–159 mmHg or DBP 90–99 mmHg | 25 |
|  |  | - SBP ≥160 mmHg or DBP ≥100 mmHg | 0 |
|  | Blood Glucose | - Fasting glucose <100 mg/dL, no history of diabetes | 100 |
|  |  | - Fasting glucose 100–125 mg/dL, no history of diabetes | 60 |
|  |  | - Fasting glucose ≥126 mg/dL or diabetes | 0 |
|  | BMI | - <25.0 kg/m² | 100 |
|  |  | - 25.0–29.9 kg/m² | 70 |
|  |  | - 30.0–34.9 kg/m² | 30 |
|  |  | - 35.0–39.9 kg/m² | 15 |
|  |  | - ≥40.0 kg/m² | 0 |
| Health Behaviors | Diet Quality (HEI-2015 score percentile) | - ≥95th percentile | 100 |
|  |  | - 75th–94th percentile | 80 |
|  |  | - 50th–74th percentile | 50 |
|  |  | - 25th–49th percentile | 25 |
|  |  | - 1st–24th percentile | 0 |
|  | Physical Activity | - ≥150 min/week of moderate or vigorous activity | 100 |
|  |  | - 120–149 min/week | 90 |
|  |  | - 90–119 min/week | 80 |
|  |  | - 60–89 min/week | 60 |
|  |  | - 30–59 min/week | 40 |
|  |  | - 1–29 min/week | 20 |
|  |  | - 0 min/week | 0 |
|  | Sleep Duration | - 7 to <9 hours/night | 100 |
|  |  | - 9 to <10 hours/night | 90 |
|  |  | - 6 to <7 hours/night | 70 |
|  |  | - 5 to <6 hours/night or ≥10 hours/night | 40 |
|  |  | - 4 to <5 hours/night | 20 |
|  |  | - <4 hours/night | 0 |

**Abbreviations:** SBP, systolic blood pressure; DBP, diastolic blood pressure; BMI, body mass index; HEI-2015, Healthy Eating Index 2015. **Note:** We did not include nicotine exposure and blood lipids in the LE8 calculation due to unavailability of data.

**Supplemental Figure S1.** Differentially methylated positions (DMPs) and regions (DMRs) in human skeletal muscle associated with walking speed.

(a) Manhattan plots showing epigenome-wide associations. Labelled are top 10 significant DMPs (five hypermethylated and five hypomethylated), ranked by beta coefficients. The dashed blue line indicates the statistical significance threshold (BACON-corrected FDR<0.05), while the red line represents the suggestive significance threshold (BACON-corrected FDR<0.1). (b) Annotations of significant DMPs by CpG island context and genomic context, with hypermethylated in red and hypomethylated in teal. (c) Annotations of significant DMRs by genomic context, with hypermethylated in red and hypomethylated in teal. (d) Venn diagram showing the number of differentially methylated genes mapped from DMPs and DMRs.

**Supplemental Figure S2.** Differentially methylated positions (DMPs) and regions (DMRs) in human skeletal muscle associated with handgrip strength.

(a) Manhattan plots showing epigenome-wide associations. The dashed blue line indicates the statistical significance threshold (BACON-corrected FDR<0.05), while the red line represents the suggestive significance threshold (BACON-corrected FDR<0.1). (b) Annotations of significant DMRs by CpG island context and genomic context, with hypermethylated in red and hypomethylated in teal. (c) Venn diagram showing the number of differentially methylated genes mapped from DMRs. No significant DMPs were identified.

**Supplemental Figure S3.** Differentially methylated positions (DMPs) and regions (DMRs) in human skeletal muscle associated with perceived disability.

(a) Manhattan plots showing epigenome-wide associations. Labelled are top 10 significant DMPs (five hypermethylated and five hypomethylated), ranked by beta coefficients. The dashed blue line indicates the statistical significance threshold (BACON-corrected FDR<0.05), while the red line represents the suggestive significance threshold (BACON-corrected FDR<0.1). (b) Annotations of significant DMPs by CpG island context and genomic context, with hypermethylated in red and hypomethylated in teal. (c) Annotations of significant DMRs by genomic context, with hypermethylated in red and hypomethylated in teal. (d) Venn diagram showing the number of differentially methylated genes mapped from DMPs and DMRs.

**Supplemental Figure S4.** Differentially methylated positions (DMPs) and regions (DMRs) in human skeletal muscle associated with cardiovascular and lifestyle health.

(a) Manhattan plots showing epigenome-wide associations. Labelled are top 10 significant DMPs (five hypermethylated and five hypomethylated), ranked by beta coefficients. The dashed blue line indicates the statistical significance threshold (BACON-corrected FDR<0.05), while the red line represents the suggestive significance threshold (BACON-corrected FDR<0.1). (b) Annotations of significant DMPs by CpG island context and genomic context, with hypermethylated in red and hypomethylated in teal. (c) Venn diagram showing the number of differentially methylated genes mapped from DMPs. No significant DMRs were identified.

**Supplemental Figure S5.** Quantile–quantile (Q–Q) plots and genomic inflation factors (λ) after genomic correction.

**Supplemental Table S1.** Skeletal muscle-based DMPs associated with physical function

|  |  |  |  |  |  | Association with physical function phenotype | | | |
| --- | --- | --- | --- | --- | --- | --- | --- | --- | --- |
| CpG | Chromosome | Position | Relation to CpG islands | Gene | Gene region | Beta | SE | P | FDR |
| *SPPB score* |  |  |  |  |  |  |  |  |  |
| cg13242895 | chr1 | 216632365 | OpenSea | - | - | 0.13 | 0.03 | 5.54E-07 | 0.032 |
| cg18929316 | chr17 | 48099486 | Shore | - | - | 0.12 | 0.02 | 2.13E-08 | 0.006 |
| cg02183588 | chr22 | 47549467 | OpenSea | - | - | 0.1 | 0.02 | 1.65E-06 | 0.045 |
| cg19808663 | chr12 | 69584871 | Shore | CCT2;MIR3913-1 | TSS1500;TSS200 | 0.09 | 0.02 | 6.48E-07 | 0.032 |
| cg01287200 | chr13 | 26399977 | OpenSea | - | - | 0.09 | 0.02 | 1.28E-06 | 0.045 |
| cg13355697 | chr9 | 3469707 | OpenSea | - | - | 0.08 | 0.01 | 1.97E-07 | 0.017 |
| cg04966255 | chr8 | 141170572 | Shelf | - | - | 0.08 | 0.02 | 2.32E-06 | 0.045 |
| cg01409496 | chr14 | 78616558 | OpenSea | - | - | 0.07 | 0.01 | 8.54E-07 | 0.036 |
| cg18327695 | chr2 | 101709976 | OpenSea | - | - | 0.07 | 0.02 | 3.06E-06 | 0.045 |
| cg26262576 | chr13 | 19845888 | OpenSea | - | - | 0.06 | 0.01 | 3.62E-07 | 0.026 |
| cg26753121 | chr2 | 8437362 | OpenSea | - | - | 0.06 | 0.01 | 1.95E-06 | 0.045 |
| cg00930979 | chr7 | 93232464 | OpenSea | VPS50;HEPACAM2 | 5UTR;exon_1;TSS1500 | 0.06 | 0.01 | 2.78E-06 | 0.045 |
| cg08453449 | chr12 | 108303165 | OpenSea | - | - | 0.06 | 0.01 | 2.83E-07 | 0.023 |
| cg17318580 | chr4 | 15132956 | OpenSea | - | - | 0.06 | 0.01 | 2.67E-06 | 0.045 |
| cg08576928 | chr11 | 75148178 | OpenSea | - | - | 0.06 | 0.01 | 3.31E-06 | 0.046 |
| cg03171888 | chr19 | 5582689 | OpenSea | - | - | 0.05 | 0.01 | 3.06E-06 | 0.045 |
| cg22450406 | chr2 | 234513521 | OpenSea | - | - | 0.05 | 0.01 | 1.97E-06 | 0.045 |
| cg02922776 | chr1 | 212247290 | OpenSea | - | - | 0.05 | 0.01 | 2.47E-06 | 0.045 |
| cg02290238 | chr1 | 48777262 | Shore | BEND5 | TSS1500 | 0.05 | 0.01 | 3.00E-06 | 0.045 |
| cg26950885 | chr7 | 2647044 | Island | - | - | 0.05 | 0.01 | 3.74E-06 | 0.048 |
| cg17588491 | chr22 | 24802925 | Shelf | - | - | 0.05 | 0.01 | 6.78E-07 | 0.032 |
| cg15008085 | chr6 | 2247973 | Shelf | - | - | 0.05 | 0.01 | 2.98E-06 | 0.045 |
| cg02794674 | chr2 | 48715065 | OpenSea | - | - | 0.05 | 0.01 | 2.55E-06 | 0.045 |
| cg02650201 | chr17 | 62134596 | Shelf | - | - | 0.05 | 0.01 | 1.02E-07 | 0.015 |
| cg15285463 | chr18 | 37577551 | OpenSea | - | - | 0.05 | 0.01 | 1.38E-06 | 0.045 |
| cg07369189 | chr22 | 47388685 | OpenSea | - | - | 0.05 | 0.01 | 1.31E-08 | 0.006 |
| cg14250419 | chr9 | 125691787 | OpenSea | - | - | 0.05 | 0.01 | 2.10E-06 | 0.045 |
| cg02878119 | chr2 | 24793601 | Island | PTRHD1 | TSS1500 | 0.04 | 0.01 | 2.91E-06 | 0.045 |
| cg06966462 | chr2 | 9472678 | Shore | - | - | 0.04 | 0.01 | 1.29E-06 | 0.045 |
| cg18216010 | chr12 | 121203128 | OpenSea | - | - | 0.04 | 0.01 | 2.01E-06 | 0.045 |
| cg09952192 | chr1 | 53464184 | Shelf | - | - | 0.04 | 0.01 | 3.60E-06 | 0.047 |
| cg25880387 | chr6 | 29641993 | OpenSea | - | - | 0.04 | 0.01 | 3.53E-06 | 0.047 |
| cg20660297 | chr12 | 75512523 | Shore | KRR1 | TSS1500 | -0.14 | 0.03 | 2.58E-06 | 0.045 |
| cg25616992 | chr8 | 702342 | Shore | - | - | -0.1 | 0.02 | 2.00E-06 | 0.045 |
| cg15542016 | chr1 | 204652124 | OpenSea | - | - | -0.1 | 0.02 | 2.09E-08 | 0.006 |
| cg25326727 | chr1 | 164676015 | OpenSea | - | - | -0.09 | 0.02 | 1.12E-06 | 0.044 |
| cg11362049 | chr11 | 132101462 | OpenSea | - | - | -0.08 | 0.02 | 2.32E-06 | 0.045 |
| cg23372455 | chr19 | 6801964 | OpenSea | - | - | -0.08 | 0.02 | 4.81E-07 | 0.032 |
| cg16060733 | chr8 | 3113531 | OpenSea | - | - | -0.08 | 0.02 | 3.40E-06 | 0.046 |
| cg19647607 | chr20 | 13299299 | Island | ISM1 | exon_6 | -0.07 | 0.02 | 2.99E-06 | 0.045 |
| cg05241721 | chr6 | 28574786 | OpenSea | ZBED9 | exon_5;exon_3 | -0.07 | 0.02 | 3.27E-06 | 0.046 |
| cg04386341 | chr2 | 239355862 | OpenSea | - | - | -0.07 | 0.02 | 2.48E-06 | 0.045 |
| cg09015232 | chr20 | 46028476 | Shore | SLC12A5 | TSS1500 | -0.07 | 0.01 | 2.39E-06 | 0.045 |
| cg23066139 | chr10 | 131916614 | OpenSea | - | - | -0.07 | 0.01 | 1.87E-06 | 0.045 |
| cg04760834 | chr4 | 7380663 | OpenSea | - | - | -0.07 | 0.01 | 1.53E-06 | 0.045 |
| cg25847633 | chr6 | 35776453 | Island | CLPSL2 | TSS200 | -0.06 | 0.01 | 6.31E-07 | 0.032 |
| cg02226847 | chr12 | 130340735 | Shore | - | - | -0.06 | 0.01 | 3.05E-06 | 0.045 |
| cg19035815 | chr6 | 121614680 | OpenSea | - | - | -0.06 | 0.01 | 2.37E-06 | 0.045 |
| cg24044084 | chr4 | 169612732 | Island | NEK1 | TSS200 | -0.06 | 0.01 | 2.94E-06 | 0.045 |
| cg19516457 | chr12 | 72273927 | Shore | TRHDE-AS1 | TSS1500 | -0.06 | 0.01 | 1.42E-06 | 0.045 |
| cg21248305 | chr6 | 30164938 | OpenSea | - | - | -0.06 | 0.01 | 3.37E-06 | 0.046 |
| cg13274107 | chr2 | 101473909 | Island | - | - | -0.06 | 0.01 | 6.97E-07 | 0.032 |
| cg26343043 | chr22 | 39228063 | Shelf | - | - | -0.06 | 0.01 | 2.45E-06 | 0.045 |
| cg17826344 | chr19 | 50666403 | Island | SHANK1 | exon_23 | -0.05 | 0.01 | 7.60E-07 | 0.033 |
| cg13272809 | chr3 | 109225077 | OpenSea | - | - | -0.05 | 0.01 | 3.84E-06 | 0.048 |
| cg26661257 | chr14 | 61696373 | Island | HIF1A;HIF1A-AS1 | TSS1500 | -0.05 | 0.01 | 2.79E-06 | 0.045 |
| cg23643660 | chr15 | 81331341 | OpenSea | TMC3 | 3UTR;exon_22 | -0.05 | 0.01 | 1.30E-06 | 0.045 |
| cg25121831 | chr1 | 36081074 | Shelf | - | - | -0.05 | 0.01 | 1.25E-07 | 0.015 |
| cg21564365 | chr2 | 235811232 | OpenSea | - | - | -0.05 | 0.01 | 2.64E-06 | 0.045 |
| cg19724680 | chr14 | 49863183 | Island | RN7SL2 | TSS1500 | -0.05 | 0.01 | 2.82E-08 | 0.006 |
| cg23228491 | chr7 | 55952318 | Shore | - | - | -0.05 | 0.01 | 2.11E-06 | 0.045 |
| cg04288036 | chr11 | 62792241 | Shore | NXF1;TMEM223 | 3UTR;exon_20;exon_21;TSS1500 | -0.05 | 0.01 | 1.59E-07 | 0.015 |
| cg25788116 | chr21 | 32569649 | Island | - | - | -0.05 | 0.01 | 6.07E-08 | 0.011 |
| cg08188248 | chr5 | 80570803 | Shore | ANKRD34B | TSS1500 | -0.04 | 0.01 | 3.81E-06 | 0.048 |
| cg00409965 | chr16 | 31033047 | Island | STX4 | TSS1500;TSS200 | -0.04 | 0.01 | 2.04E-06 | 0.045 |
| cg14817043 | chr20 | 2693583 | Island | EBF4 | 5UTR;exon_1 | -0.04 | 0.01 | 6.13E-07 | 0.032 |
| cg20977327 | chr1 | 248826260 | Island | MIR3124;SH3BP5L | TSS200;TSS1500 | -0.04 | 0.01 | 1.45E-07 | 0.015 |
| cg09837632 | chr2 | 215311992 | Island | ATIC | TSS200 | -0.04 | 0.01 | 1.66E-06 | 0.045 |
| cg20056914 | chr14 | 23042846 | OpenSea | PSMB11 | exon_1 | -0.04 | 0.01 | 3.40E-06 | 0.046 |
| cg11332994 | chr5 | 70049314 | Shore | SMN2 | TSS1500 | -0.03 | 0.01 | 1.51E-06 | 0.045 |
| *6MWT walking speed* | |  |  |  |  |  |  |  |  |
| cg27592988 | chr7 | 128707747 | OpenSea | FAM71F1 | TSS1500 | 1.16 | 0.18 | 8.94E-11 | 7.83E-05 |
| cg17308762 | chr8 | 41803604 | OpenSea | - | - | 1.07 | 0.22 | 7.41E-07 | 0.041 |
| cg25886345 | chr4 | 68311147 | OpenSea | YTHDC1 | 3UTR;exon_17;exon_16 | 1.03 | 0.2 | 2.09E-07 | 0.03 |
| cg16037140 | chr11 | 13215319 | OpenSea | - | - | 1.01 | 0.18 | 9.34E-09 | 0.003 |
| cg24701190 | chr10 | 118914351 | OpenSea | - | - | 0.94 | 0.19 | 4.12E-07 | 0.039 |
| cg10957528 | chr7 | 23532992 | Shore | TRA2A | TSS1500 | 0.77 | 0.14 | 3.98E-08 | 0.007 |
| cg04083035 | chr1 | 116471687 | OpenSea | - | - | 0.75 | 0.15 | 4.97E-07 | 0.039 |
| cg10540364 | chr10 | 21494610 | Island | MIR1915HG | exon_2 | 0.66 | 0.13 | 6.22E-07 | 0.039 |
| cg06555729 | chr15 | 99384075 | OpenSea | - | - | 0.66 | 0.13 | 7.22E-07 | 0.041 |
| cg06972967 | chr16 | 66994504 | OpenSea | - | - | 0.65 | 0.13 | 5.54E-07 | 0.039 |
| cg07921851 | chr10 | 110455188 | OpenSea | - | - | 0.48 | 0.09 | 4.31E-07 | 0.039 |
| cg15554005 | chr11 | 128871418 | OpenSea | - | - | 0.45 | 0.08 | 1.46E-08 | 0.003 |
| cg07331460 | chr7 | 156116138 | OpenSea | - | - | -0.74 | 0.15 | 6.05E-07 | 0.039 |
| cg02775617 | chr10 | 31928353 | Island | - | - | -0.65 | 0.13 | 6.17E-07 | 0.039 |
| cg07696690 | chr16 | 20900256 | Island | DCUN1D3;LYRM1 | 5UTR;exon_1;TSS1500;TSS200 | -0.62 | 0.12 | 6.20E-07 | 0.039 |
| cg19062112 | chr14 | 102592717 | Island | RCOR1 | 5UTR;exon_1 | -0.54 | 0.09 | 7.26E-09 | 0.003 |
| *Perceived disability* | |  |  |  |  |  |  |  |  |
| cg02867811 | chr6 | 45327631 | OpenSea | RUNX2 | TSS1500 | 1.91 | 0.35 | 3.52E-08 | 0.008 |
| cg08264579 | chr5 | 90408016 | Shore | - | - | 1.45 | 0.28 | 3.13E-07 | 0.045 |
| cg24938788 | chr20 | 6299417 | OpenSea | - | - | 1.26 | 0.21 | 2.01E-09 | 0.001 |
| cg13033853 | chr9 | 130939012 | Island | FIBCD1 | 5UTR;exon_1 | 1.18 | 0.17 | 1.39E-11 | 1.21E-05 |
| cg13948486 | chr2 | 162260663 | OpenSea | - | - | 1.15 | 0.23 | 7.47E-07 | 0.045 |
| cg14306821 | chr9 | 128984927 | OpenSea | NUP188 | exon_20 | 0.75 | 0.15 | 7.66E-07 | 0.045 |
| cg05511326 | chr3 | 129662169 | OpenSea | - | - | 0.54 | 0.11 | 7.24E-07 | 0.045 |
| cg25151919 | chr3 | 44712841 | Island | - | - | 0.34 | 0.07 | 1.06E-06 | 0.049 |
| cg14477266 | chr12 | 118052650 | OpenSea | - | - | -0.9 | 0.18 | 9.91E-07 | 0.048 |
| cg17809941 | chr11 | 74237605 | OpenSea | - | - | -0.72 | 0.13 | 2.04E-08 | 0.006 |
| cg00369590 | chr11 | 70744064 | OpenSea | - | - | -0.57 | 0.12 | 8.19E-07 | 0.045 |
| cg22869599 | chr7 | 4235751 | OpenSea | - | - | -0.55 | 0.11 | 9.53E-07 | 0.048 |
| cg13149730 | chr21 | 46315066 | OpenSea | - | - | -0.49 | 0.1 | 7.20E-07 | 0.045 |
| cg22460512 | chr11 | 83312166 | OpenSea | - | - | -0.46 | 0.09 | 2.93E-07 | 0.045 |
| cg03901910 | chr8 | 1640733 | Shore | - | - | -0.44 | 0.09 | 6.14E-07 | 0.045 |
| cg10502963 | chr7 | 379946 | Island | - | - | -0.4 | 0.08 | 6.25E-07 | 0.045 |
| cg12330031 | chr22 | 49930490 | Shore | - | - | -0.37 | 0.07 | 5.00E-07 | 0.045 |
| cg16013757 | chr11 | 10583420 | OpenSea | - | - | -0.31 | 0.06 | 5.69E-07 | 0.045 |
| cg09870201 | chr20 | 51378294 | OpenSea | - | - | -0.3 | 0.06 | 7.65E-07 | 0.045 |
| *Cardiovascular and lifestyle score* | | |  |  |  |  |  |  |  |
| cg23530586 | chr5 | 100595733 | OpenSea | - | - | 0.01 | 0 | 9.23E-09 | 0.008 |
| cg01817084 | chr8 | 26292049 | OpenSea | PPP2R2A | TSS1500 | -0.01 | 0 | 1.05E-07 | 0.046 |

Abbreviations: DMP, differentially methylated position; SPPB, Short Physical Performance Battery; 6MWT, 6-Minute Walk Test.

**Supplemental Table S2.** Skeletal muscle-based DMRs associated with physical function

|  |  |  |  |  | **Association with physical function phenotype** | |
| --- | --- | --- | --- | --- | --- | --- |
| **Chromosome** | **Genomic Coordinates** | **N CpGs** | **Gene** | **Gene region** | **Mean beta** | **Combined FDR** |
| ***SPPB score*** | |  |  |  |  |  |
| chr11 | 67605481-67605643 | 3 | NDUFV1 | TSS1500 | 0.012 | 0.008 |
| chr22 | 45226922-45227185 | 4 | - | - | 0.009 | 2.88E-04 |
| chr1 | 44721879-44722274 | 5 | RNU5F-1 | TSS1500;exon_1;TSS200 | 0.008 | 0.001 |
| chr1 | 3658140-3658435 | 3 | - | - | 0.007 | 3.20E-05 |
| chr6 | 30743784-30744025 | 10 | IER3;FLOT1 | 3UTR;exon_2;TSS1500 | 0.006 | 0.017 |
| chr5 | 103562449-103563257 | 11 | NUDT12 | TSS200;5UTR;exon_1;TSS1500 | 0.006 | 0.006 |
| chr16 | 67943962-67944547 | 10 | LCAT;SLC12A4 | TSS200;3UTR;exon_24;exon_23;exon_1;TSS1500 | 0.006 | 3.71E-04 |
| chr5 | 138875060-138875495 | 9 | LRRTM2;CTNNA1 | exon_1;5UTR;TSS1500;TSS200 | 0.005 | 0.008 |
| chr12 | 108302785-108303505 | 4 | - | - | 0.005 | 3.10E-06 |
| chr10 | 88952868-88953250 | 7 | ACTA2 | TSS1500;TSS200 | 0.004 | 0.001 |
| chr8 | 141222853-141223606 | 6 | - | - | 0.004 | 4.55E-05 |
| chr10 | 104274714-104275410 | 11 | GSTO2 | TSS200;5UTR;exon_2;TSS1500 | 0.003 | 0.002 |
| chr1 | 3650844-3651681 | 18 | TP73;WRAP73 | TSS1500 | 0.003 | 0.01 |
| chr6 | 31740449-31740735 | 6 | MSH5;MSH5-SAPCD1 | exon_2 | 0.002 | 4.44E-05 |
| chr17 | 1604349-1605791 | 11 | SLC43A2 | exon_1;5UTR;TSS1500;TSS200 | -0.01 | 0.002 |
| chr22 | 29313975-29314542 | 4 | RASL10A | exon_2 | -0.007 | 2.14E-04 |
| chr5 | 81301324-81302009 | 13 | CKMT2-AS1;ZCCHC9 | exon_1;TSS200;5UTR;TSS1500 | -0.006 | 0.002 |
| chr1 | 101847052-101847115 | 3 | OLFM3 | TSS200 | -0.005 | 0.001 |
| chr17 | 43125677-43126889 | 14 | BRCA1;NBR2 | TSS1500;exon_1 | -0.005 | 0.001 |
| chr6 | 28615878-28616687 | 17 | ZBED9 | TSS200;TSS1500;5UTR;exon_1 | -0.004 | 0.001 |
| chr6 | 32095776-32097065 | 31 | TNXB | exon_3 | -0.004 | 0.011 |
| chr22 | 31662133-31662824 | 10 | PISD | exon_1;5UTR;TSS200;TSS1500 | -0.003 | 2.88E-04 |
| ***6MWT walking speed*** | |  |  |  |  |  |
| chr5 | 136079169-136080924 | 19 | VTRNA2-1 | TSS200;TSS1500;exon_1 | 0.126 | 1.85E-04 |
| chr1 | 114099885-114100239 | 3 | - | - | 0.114 | 3.36E-04 |
| chr18 | 74498194-74499978 | 8 | CNDP2 | exon_1;exon_2;exon_4;5UTR;exon_3;TSS1500;TSS200 | 0.111 | 8.61E-05 |
| chr2 | 113235117-113235736 | 8 | PAX8-AS1;PAX8 | TSS1500;exon_9;TSS200;exon_1 | 0.109 | 0.018 |
| chr2 | 3605056-3605893 | 6 | COLEC11 | TSS1500;TSS200 | 0.105 | 0.001 |
| chr2 | 130288549-130288978 | 4 | - | - | 0.104 | 0.002 |
| chr20 | 32792069-32792762 | 4 | DNMT3B | exon_8;exon_7;exon_9 | 0.101 | 0.001 |
| chr12 | 50222317-50222479 | 3 | LIMA1 | exon_4;exon_1;5UTR;exon_5 | 0.099 | 0.02 |
| chr15 | 39976543-39977013 | 3 | EIF2AK4 | exon_12 | 0.097 | 0.01 |
| chr1 | 55944024-55944335 | 3 | LINC01755 | TSS1500 | 0.096 | 0.003 |
| chr17 | 83103203-83105373 | 14 | - | - | 0.095 | 0.005 |
| chr2 | 240983361-240983938 | 4 | - | - | 0.086 | 0.005 |
| chr16 | 86578084-86578845 | 8 | FOXL1 | exon_1;5UTR;TSS1500;TSS200 | 0.086 | 0.007 |
| chr11 | 14905234-14906003 | 5 | - | - | 0.085 | 2.97E-04 |
| chr13 | 99565670-99566759 | 7 | - | - | 0.083 | 0.001 |
| chr4 | 173535353-173536793 | 6 | - | - | 0.081 | 4.67E-04 |
| chr22 | 45226922-45227185 | 4 | - | - | 0.08 | 0.001 |
| chr11 | 58424250-58424591 | 3 | - | - | 0.08 | 9.89E-05 |
| chr3 | 15287391-15287806 | 4 | - | - | 0.079 | 0.001 |
| chr5 | 1413268-1413905 | 3 | - | - | 0.078 | 0.001 |
| chr18 | 57622565-57622593 | 3 | NARS1 | TSS1500 | 0.077 | 0.001 |
| chr13 | 99316862-99317019 | 3 | - | - | 0.073 | 1.26E-04 |
| chr2 | 176158234-176159566 | 9 | - | - | 0.073 | 0.011 |
| chr14 | 91396469-91396563 | 4 | - | - | 0.073 | 0.002 |
| chr2 | 176163128-176164731 | 11 | HOXD3 | 5UTR;exon_2 | 0.072 | 0.01 |
| chr8 | 29905341-29905618 | 3 | - | - | 0.072 | 1.57E-04 |
| chr18 | 63489832-63490606 | 6 | - | - | 0.07 | 0.001 |
| chr2 | 176123475-176124211 | 6 | HOXD9 | exon_1;exon_2;3UTR | 0.07 | 0.01 |
| chr15 | 100910487-100910634 | 3 | - | - | 0.069 | 3.94E-04 |
| chr11 | 78561221-78561697 | 3 | - | - | 0.068 | 3.68E-05 |
| chr7 | 151590759-151590951 | 3 | - | - | 0.066 | 0.002 |
| chr10 | 21509769-21510466 | 4 | - | - | 0.065 | 0.001 |
| chr6 | 169577298-169578291 | 5 | - | - | 0.064 | 0.002 |
| chr7 | 129169259-129169601 | 5 | TSPAN33 | exon_8;3UTR | 0.063 | 0.005 |
| chr15 | 40106798-40106900 | 3 | BMF | TSS1500;TSS200 | 0.063 | 0.002 |
| chr4 | 84503042-84503316 | 3 | - | - | 0.062 | 0.001 |
| chr21 | 35048967-35049815 | 9 | RUNX1 | 5UTR;exon_1;TSS1500;TSS200 | 0.062 | 2.40E-04 |
| chr17 | 80891287-80892554 | 10 | RPTOR | exon_14;exon_18 | 0.061 | 0.003 |
| chr1 | 2922240-2922655 | 5 | - | - | 0.06 | 0.001 |
| chr3 | 128142045-128142208 | 4 | - | - | 0.059 | 0.001 |
| chr7 | 102933787-102934057 | 3 | LRRC17 | exon_2;5UTR | 0.059 | 0.003 |
| chr2 | 20595685-20596152 | 3 | - | - | 0.059 | 5.59E-05 |
| chr3 | 128075376-128076551 | 9 | RUVBL1-AS1 | TSS1500;TSS200;exon_1 | 0.058 | 0.001 |
| chr19 | 822842-823856 | 4 | PLPPR3 | TSS1500 | 0.058 | 3.50E-04 |
| chr1 | 107139554-107139680 | 3 | NTNG1 | TSS1500 | 0.058 | 0.002 |
| chr13 | 52739760-52740091 | 5 | CNMD | exon_1;5UTR;TSS1500;TSS200 | 0.057 | 0.007 |
| chr10 | 76051269-76051662 | 3 | - | - | 0.056 | 0.001 |
| chr3 | 137642684-137643086 | 3 | - | - | 0.056 | 3.99E-05 |
| chr22 | 50547252-50548533 | 7 | KLHDC7B | exon_1 | 0.056 | 0.001 |
| chr6 | 158626112-158626333 | 4 | - | - | 0.056 | 0.001 |
| chr2 | 176149281-176153069 | 33 | HOXD4;MIR10B | TSS1500;TSS200;exon_2;exon_1;3UTR | 0.056 | 0.006 |
| chr2 | 9631939-9632246 | 3 | YWHAQ | TSS1500 | 0.056 | 0.001 |
| chr14 | 58919298-58919546 | 3 | - | - | 0.056 | 1.95E-05 |
| chr1 | 63012361-63012500 | 3 | LINC01739 | exon_1;TSS1500;TSS200 | 0.056 | 4.29E-04 |
| chr1 | 153549183-153549525 | 6 | S100A3 | 5UTR;exon_1;TSS200;TSS1500 | 0.055 | 0.008 |
| chr22 | 45503412-45503856 | 3 | - | - | 0.055 | 0.002 |
| chr1 | 3771240-3771693 | 4 | CCDC27;SMIM1 | exon_12;3UTR;TSS1500 | 0.054 | 0.002 |
| chr11 | 114294939-114296970 | 9 | NNMT | TSS1500;TSS200 | 0.054 | 1.18E-05 |
| chr5 | 37835446-37835599 | 4 | GDNF | TSS1500;TSS200;5UTR;exon_1 | 0.053 | 0.026 |
| chr4 | 8392440-8392970 | 5 | ACOX3 | exon_11;exon_10 | 0.053 | 0.004 |
| chr10 | 71766472-71766907 | 3 | - | - | 0.053 | 8.74E-05 |
| chr17 | 59840901-59841269 | 4 | VMP1;MIR21 | exon_12;3UTR;exon_13;exon_11;exon_10;TSS200;exon_1;TSS1500 | 0.053 | 3.54E-04 |
| chr1 | 119711554-119711707 | 4 | PHGDH | TSS1500 | 0.053 | 9.40E-05 |
| chr16 | 87065518-87066623 | 5 | - | - | 0.052 | 2.91E-04 |
| chr19 | 6730702-6730723 | 3 | - | - | 0.052 | 3.52E-04 |
| chr10 | 131260594-131260725 | 4 | - | - | 0.052 | 4.17E-04 |
| chr9 | 127353865-127354139 | 3 | GARNL3 | exon_19;exon_18 | 0.052 | 0.002 |
| chr4 | 171639462-171639642 | 3 | LINC02174 | TSS1500 | 0.051 | 2.56E-04 |
| chr1 | 91736928-91737430 | 4 | - | - | 0.051 | 1.57E-04 |
| chr2 | 44949844-44950104 | 3 | - | - | 0.051 | 1.78E-04 |
| chr4 | 151227670-151227912 | 3 | SH3D19 | TSS1500;TSS200;5UTR;exon_1 | 0.051 | 0.008 |
| chr1 | 201648218-201648725 | 6 | NAV1 | TSS1500;exon_5;exon_4;exon_1 | 0.051 | 0.02 |
| chr11 | 59811041-59811202 | 4 | MRPL16 | TSS1500 | 0.05 | 0.001 |
| chr7 | 73742887-73743687 | 5 | - | - | 0.05 | 0.001 |
| chr1 | 83860502-83861743 | 12 | LOC101927560;LINC01725 | TSS200;exon_1;TSS1500 | 0.05 | 0.001 |
| chr10 | 131616652-131617753 | 6 | - | - | 0.05 | 0.003 |
| chr5 | 83720811-83721365 | 8 | HAPLN1 | TSS1500;5UTR;exon_1;TSS200 | 0.049 | 0.004 |
| chr16 | 14370358-14370518 | 4 | LINC02130 | TSS200;TSS1500 | 0.049 | 2.52E-04 |
| chr6 | 30154746-30155684 | 9 | - | - | 0.049 | 0.005 |
| chr15 | 77144143-77144783 | 4 | - | - | 0.049 | 0.002 |
| chr16 | 67277628-67278010 | 3 | PLEKHG4 | TSS1500;TSS200 | 0.049 | 0.003 |
| chr16 | 87044980-87045707 | 5 | - | - | 0.049 | 0.002 |
| chr2 | 112541078-112541373 | 3 | TTL;POLR1B | exon_7;3UTR;TSS1500 | 0.049 | 2.69E-04 |
| chr3 | 46406335-46406643 | 3 | CCRL2 | TSS1500 | 0.049 | 1.59E-04 |
| chr14 | 61546612-61547298 | 3 | - | - | 0.048 | 3.39E-04 |
| chr10 | 92690299-92692723 | 17 | HHEX | exon_1;exon_2;exon_3 | 0.048 | 3.04E-04 |
| chr8 | 681217-682172 | 3 | - | - | 0.048 | 2.29E-05 |
| chr1 | 156149629-156150188 | 5 | SEMA4A | TSS1500;TSS200 | 0.048 | 0.008 |
| chr16 | 30473975-30474645 | 6 | ITGAL | exon_2 | 0.048 | 0.003 |
| chr4 | 173528337-173528865 | 6 | HAND2-AS1;HAND2 | exon_1;TSS1500;TSS200 | 0.048 | 0.006 |
| chr21 | 42562055-42562450 | 5 | SLC37A1 | exon_12;exon_13 | 0.048 | 0.005 |
| chr12 | 51392254-51393018 | 4 | GALNT6 | TSS1500 | 0.048 | 0.003 |
| chr19 | 55537890-55538457 | 4 | SBK2 | TSS1500 | 0.047 | 2.83E-04 |
| chr8 | 120125894-120126078 | 3 | - | - | 0.047 | 0.001 |
| chr11 | 112166379-112166950 | 5 | TEX12 | TSS1500 | 0.046 | 2.58E-04 |
| chr9 | 135700643-135701165 | 3 | SOHLH1;KCNT1 | TSS1500 | 0.046 | 1.50E-04 |
| chr10 | 99233268-99233905 | 9 | - | - | 0.045 | 0.025 |
| chr2 | 3656398-3657681 | 8 | ALLC | TSS1500 | 0.045 | 4.52E-04 |
| chr16 | 66842944-66843035 | 3 | CA7 | TSS1500 | 0.045 | 0.002 |
| chr20 | 32806322-32806498 | 3 | DNMT3B | exon_22;exon_21 | 0.045 | 0.002 |
| chr1 | 6520050-6520465 | 6 | PLEKHG5 | TSS200;TSS1500;5UTR;exon_1 | 0.045 | 0.002 |
| chr1 | 3940065-3940684 | 8 | LINC01346 | TSS1500;TSS200;exon_1 | 0.044 | 0.002 |
| chr11 | 13215319-13216653 | 4 | - | - | 0.044 | 6.58E-07 |
| chr17 | 12990072-12990331 | 3 | ARHGAP44 | 3UTR;exon_21;exon_20;exon_22;exon_18 | 0.044 | 2.85E-05 |
| chr15 | 61365143-61365271 | 3 | - | - | 0.044 | 0.001 |
| chr8 | 1989401-1989757 | 4 | - | - | 0.044 | 0.001 |
| chr5 | 1003389-1004664 | 7 | - | - | 0.043 | 0.001 |
| chr16 | 81899378-81899865 | 4 | - | - | 0.043 | 1.01E-04 |
| chr6 | 75202000-75202468 | 5 | - | - | 0.043 | 0.007 |
| chr8 | 26041323-26041741 | 4 | - | - | 0.043 | 0.009 |
| chr7 | 46969297-46969428 | 3 | - | - | 0.043 | 0.001 |
| chr3 | 128553880-128554278 | 3 | - | - | 0.043 | 1.77E-04 |
| chr4 | 8228326-8229120 | 6 | SH3TC1 | exon_12;exon_10 | 0.043 | 0.001 |
| chr22 | 37735414-37736090 | 3 | TRIOBP | exon_9 | 0.042 | 3.87E-04 |
| chr22 | 43516029-43516199 | 4 | EFCAB6-AS1 | TSS200;TSS1500 | 0.042 | 0.001 |
| chr10 | 117131466-117132195 | 7 | VAX1 | 3UTR;exon_4 | 0.042 | 0.006 |
| chr10 | 121263166-121263268 | 4 | - | - | 0.042 | 6.66E-05 |
| chr10 | 76112326-76112858 | 4 | - | - | 0.042 | 0.002 |
| chr19 | 1065185-1066350 | 10 | ARHGAP45;ABCA7 | TSS1500;TSS200;exon_47 | 0.042 | 0.001 |
| chr18 | 54827850-54828173 | 4 | RAB27B | TSS1500 | 0.042 | 2.06E-04 |
| chr11 | 43920385-43921132 | 6 | ALKBH3-AS1 | exon_1;TSS200 | 0.041 | 3.31E-04 |
| chr10 | 132124389-132124702 | 3 | - | - | 0.041 | 0.001 |
| chr21 | 45544429-45544735 | 4 | - | - | 0.041 | 0.006 |
| chr19 | 44613396-44613597 | 5 | IGSF23 | TSS200;5UTR;exon_1 | 0.041 | 0.001 |
| chr18 | 78543815-78544675 | 6 | - | - | 0.041 | 3.87E-04 |
| chr2 | 190406794-190407838 | 5 | MFSD6 | TSS1500;TSS200 | 0.04 | 0.001 |
| chr5 | 176135111-176136016 | 3 | - | - | 0.04 | 2.85E-05 |
| chr15 | 63596321-63597072 | 5 | FBXL22 | TSS1500 | 0.04 | 0.003 |
| chr8 | 23572480-23573501 | 5 | SLC25A37 | exon_4;3UTR;exon_5 | 0.04 | 2.18E-04 |
| chr19 | 8392452-8392875 | 3 | - | - | 0.039 | 1.90E-04 |
| chr1 | 155972294-155972551 | 4 | - | - | 0.039 | 0.001 |
| chr11 | 119920940-119921107 | 3 | - | - | 0.039 | 6.15E-05 |
| chr5 | 180138041-180138362 | 3 | RASGEF1C | exon_2 | 0.039 | 0.001 |
| chr19 | 2550867-2551327 | 3 | - | - | 0.039 | 1.89E-05 |
| chr17 | 37745038-37745373 | 8 | HNF1B | TSS1500;TSS200;exon_1;5UTR | 0.039 | 0.006 |
| chr11 | 134503266-134503733 | 5 | - | - | 0.039 | 2.46E-04 |
| chr12 | 112410854-112411333 | 3 | RPL6 | TSS1500;5UTR;exon_2;exon_1 | 0.039 | 0.001 |
| chr4 | 173510579-173510771 | 4 | - | - | 0.039 | 0.002 |
| chr5 | 61265824-61266057 | 3 | LINC02057 | TSS1500 | 0.039 | 0.001 |
| chr9 | 33443167-33443979 | 3 | AQP3 | exon_3 | 0.039 | 3.74E-04 |
| chr16 | 87067855-87068441 | 7 | - | - | 0.039 | 0.017 |
| chr11 | 118636455-118636726 | 3 | - | - | 0.038 | 0.001 |
| chr8 | 42843793-42844140 | 4 | THAP1 | TSS1500 | 0.038 | 1.81E-04 |
| chr14 | 95312370-95312815 | 3 | - | - | 0.038 | 2.29E-04 |
| chr22 | 20966704-20967775 | 7 | AIFM3 | TSS1500;5UTR;exon_1;TSS200 | 0.038 | 3.64E-04 |
| chr8 | 141222853-141223606 | 6 | - | - | 0.038 | 5.63E-05 |
| chr1 | 190474765-190475536 | 4 | - | - | 0.038 | 1.54E-04 |
| chr6 | 101398197-101400360 | 21 | GRIK2 | 5UTR;exon_1;exon_2;TSS200;TSS1500 | 0.038 | 0.005 |
| chr16 | 4371097-4371653 | 8 | VASN | TSS1500;TSS200 | 0.038 | 0.003 |
| chr3 | 48371400-48372217 | 4 | FBXW12 | TSS200;TSS1500 | 0.038 | 4.67E-05 |
| chr22 | 30408837-30409228 | 5 | SEC14L2 | exon_4;exon_5;exon_6 | 0.037 | 0.001 |
| chr22 | 37904594-37905229 | 4 | MICALL1 | TSS1500 | 0.037 | 0.002 |
| chr4 | 40514623-40514874 | 4 | RBM47 | TSS1500;TSS200 | 0.037 | 3.17E-04 |
| chr1 | 95139959-95140628 | 3 | - | - | 0.037 | 1.79E-04 |
| chr6 | 31772060-31772514 | 3 | - | - | 0.037 | 0.001 |
| chr11 | 37278555-37278853 | 3 | - | - | 0.037 | 2.05E-04 |
| chr18 | 35355340-35355771 | 3 | - | - | 0.037 | 9.85E-05 |
| chr4 | 4861951-4863175 | 15 | MSX1 | exon_2;3UTR | 0.037 | 0.012 |
| chr7 | 151012098-151012448 | 3 | ATG9B;NOS3 | exon_18;exon_17;exon_24 | 0.036 | 0.003 |
| chr11 | 6497033-6497347 | 10 | DNHD1 | TSS1500;TSS200;5UTR;exon_1 | 0.036 | 0.011 |
| chr10 | 84540831-84541196 | 3 | - | - | 0.036 | 1.09E-04 |
| chr13 | 114115286-114116081 | 8 | - | - | 0.036 | 2.21E-04 |
| chr21 | 42367362-42367716 | 4 | TFF1 | TSS1500 | 0.036 | 0.001 |
| chr22 | 50012625-50012985 | 5 | IL17REL | TSS1500;5UTR;exon_1;TSS200 | 0.036 | 0.005 |
| chr1 | 58703585-58704253 | 5 | - | - | 0.036 | 0.002 |
| chr12 | 75390761-75391515 | 11 | GLIPR1L2;CAPS2 | TSS200;exon_1;TSS1500;5UTR | 0.036 | 0.009 |
| chr2 | 69770093-69770464 | 3 | - | - | 0.036 | 3.46E-04 |
| chr18 | 77248673-77249538 | 16 | GALR1 | TSS1500 | 0.036 | 0.006 |
| chr16 | 86565186-86565862 | 5 | FOXC2;FOXC2-AS1 | TSS1500;exon_2 | 0.035 | 3.88E-04 |
| chr5 | 74684214-74684591 | 3 | HEXB | TSS1500 | 0.035 | 3.46E-05 |
| chr5 | 138874861-138875060 | 5 | LRRTM2;CTNNA1 | exon_1;5UTR;TSS1500;TSS200 | 0.035 | 1.00E-04 |
| chr15 | 90929937-90930281 | 7 | UNC45A;HDDC3 | 5UTR;exon_1;3UTR;exon_4;TSS200;TSS1500 | 0.035 | 0.003 |
| chr1 | 32881627-32882514 | 4 | - | - | 0.035 | 3.40E-05 |
| chr18 | 32772542-32773736 | 11 | KLHL14 | TSS200;TSS1500;5UTR;exon_1 | 0.035 | 0.012 |
| chr19 | 51724056-51724795 | 6 | HAS1 | TSS200;TSS1500 | 0.035 | 0.001 |
| chr3 | 141367978-141368521 | 6 | ZBTB38 | TSS200;5UTR;exon_1;TSS1500 | 0.035 | 0.005 |
| chr11 | 117824657-117825300 | 6 | FXYD2 | TSS200;TSS1500;exon_1 | 0.035 | 0.001 |
| chr7 | 1326109-1326232 | 3 | - | - | 0.035 | 0.004 |
| chr1 | 22965290-22965673 | 3 | LACTBL1 | exon_3 | 0.035 | 2.61E-04 |
| chr12 | 120314325-120314467 | 3 | - | - | 0.035 | 5.10E-05 |
| chr19 | 41362592-41363504 | 3 | TMEM91;B9D2 | TSS1500;exon_2 | 0.034 | 1.39E-05 |
| chr17 | 35448760-35449664 | 10 | SLFN13 | TSS200;TSS1500;5UTR;exon_1 | 0.034 | 0.003 |
| chr22 | 50085257-50086112 | 14 | MLC1 | exon_1;5UTR;exon_2;TSS1500;TSS200 | 0.034 | 0.002 |
| chr2 | 176112074-176113045 | 5 | - | - | 0.034 | 0.001 |
| chr3 | 15634422-15635487 | 3 | BTD | exon_2;exon_4;exon_3 | 0.034 | 5.81E-05 |
| chr12 | 2284505-2285017 | 4 | - | - | 0.034 | 3.64E-04 |
| chr1 | 79006299-79007232 | 12 | ADGRL4 | exon_1;5UTR;TSS200;TSS1500 | 0.034 | 3.51E-04 |
| chr1 | 9827753-9827926 | 3 | - | - | 0.034 | 1.84E-04 |
| chr6 | 170253240-170254459 | 4 | LOC154449 | exon_3 | 0.034 | 2.94E-05 |
| chr11 | 125082877-125083244 | 5 | - | - | 0.034 | 0.002 |
| chr15 | 81225200-81225417 | 5 | IL16 | 5UTR;exon_2 | 0.034 | 4.97E-04 |
| chr16 | 11316236-11317110 | 7 | - | - | 0.033 | 2.02E-04 |
| chr11 | 65297436-65298897 | 5 | POLA2 | exon_18;3UTR | 0.033 | 0.001 |
| chr8 | 81631184-81631659 | 3 | IMPA1P1 | exon_1;TSS1500;TSS200 | 0.033 | 0.002 |
| chr1 | 200036091-200037119 | 5 | - | - | 0.033 | 1.69E-04 |
| chr2 | 176121225-176121931 | 6 | HOXD9 | TSS1500 | 0.033 | 0.003 |
| chr3 | 11225279-11225386 | 3 | HRH1 | TSS1500 | 0.033 | 1.46E-04 |
| chr17 | 9360744-9360848 | 3 | - | - | 0.033 | 0.001 |
| chr12 | 53753977-53754499 | 5 | - | - | 0.033 | 7.76E-05 |
| chr4 | 2287077-2287849 | 4 | - | - | 0.033 | 0.001 |
| chr1 | 224457048-224457674 | 3 | - | - | 0.033 | 2.10E-05 |
| chr4 | 184001229-184001493 | 5 | STOX2 | exon_2 | 0.033 | 0.001 |
| chr16 | 4788376-4788905 | 7 | SMIM22;SEPTIN12 | 5UTR;exon_1;TSS1500;TSS200 | 0.033 | 0.01 |
| chr6 | 10418783-10419167 | 3 | - | - | 0.033 | 0.002 |
| chr1 | 35869556-35869791 | 6 | AGO1 | 5UTR;exon_1;TSS200;TSS1500 | 0.033 | 3.15E-05 |
| chr10 | 1464943-1465364 | 3 | - | - | 0.032 | 0.002 |
| chr11 | 67346273-67346658 | 3 | - | - | 0.032 | 2.56E-04 |
| chr14 | 77142127-77142800 | 4 | ZDHHC22 | TSS1500 | 0.032 | 0.004 |
| chr8 | 1676515-1676656 | 4 | DLGAP2 | exon_11 | 0.032 | 3.30E-04 |
| chr22 | 31626304-31626767 | 3 | - | - | 0.032 | 9.65E-05 |
| chr16 | 1430301-1430991 | 7 | PERCC1 | TSS1500;TSS200 | 0.032 | 0.008 |
| chr9 | 133422167-133422289 | 3 | ADAMTS13 | TSS200;TSS1500 | 0.032 | 2.29E-04 |
| chr12 | 130086701-130086797 | 3 | - | - | 0.032 | 4.26E-05 |
| chr8 | 23356449-23357045 | 6 | - | - | 0.032 | 6.20E-05 |
| chr2 | 176105780-176106627 | 5 | HOXD11 | TSS1500 | 0.032 | 0.001 |
| chr12 | 118015695-118016221 | 3 | RFC5 | TSS1500 | 0.032 | 1.84E-04 |
| chr2 | 199471710-199471957 | 3 | SATB2-AS1;SATB2 | exon_2;TSS1500 | 0.032 | 2.15E-04 |
| chr6 | 34556318-34557249 | 6 | SPDEF | TSS200;TSS1500;5UTR;exon_1 | 0.032 | 0.005 |
| chr6 | 52552058-52552312 | 4 | - | - | 0.032 | 0.001 |
| chr17 | 31094147-31094512 | 3 | NF1;MIR4733 | TSS1500;TSS200 | 0.032 | 0.004 |
| chr17 | 78979862-78980298 | 9 | LGALS3BP | TSS200;TSS1500;5UTR;exon_1 | 0.032 | 0.006 |
| chr16 | 50719277-50719362 | 3 | - | - | 0.031 | 0.001 |
| chr1 | 202189450-202189719 | 3 | - | - | 0.031 | 7.92E-05 |
| chr20 | 41340852-41341409 | 6 | LPIN3 | 5UTR;exon_1 | 0.031 | 0.009 |
| chr12 | 52010466-52010892 | 4 | TAMALIN | 5UTR;exon_1;exon_2;exon_3;TSS200 | 0.031 | 0.007 |
| chr16 | 1336074-1336289 | 4 | BAIAP3 | TSS200;5UTR;exon_1 | 0.031 | 1.63E-04 |
| chr10 | 683771-684016 | 4 | - | - | 0.031 | 0.002 |
| chr10 | 101666057-101666299 | 3 | - | - | 0.031 | 0.001 |
| chr3 | 100114449-100114949 | 3 | FILIP1L;CMSS1 | 5UTR;exon_1;TSS1500;TSS200 | 0.031 | 2.36E-04 |
| chr11 | 69470865-69471584 | 10 | - | - | 0.031 | 0.011 |
| chr10 | 44999358-45000639 | 8 | ZNF22-AS1;ZNF22 | exon_2;TSS1500;exon_1;exon_3 | 0.031 | 0.005 |
| chr20 | 54028793-54029012 | 4 | BCAS1 | exon_4 | 0.031 | 4.92E-04 |
| chr5 | 172778407-172778869 | 3 | - | - | 0.031 | 2.54E-05 |
| chr11 | 117817941-117818428 | 4 | DSCAML1 | TSS1500 | 0.031 | 0.002 |
| chr20 | 57524668-57525087 | 5 | CTCFL | 5UTR;exon_1;exon_2;TSS1500;TSS200 | 0.031 | 0.004 |
| chr17 | 83109862-83110272 | 3 | - | - | 0.031 | 4.51E-04 |
| chr4 | 15938715-15939274 | 4 | FGFBP1 | TSS200;TSS1500;5UTR;exon_1 | 0.031 | 4.74E-04 |
| chr11 | 2902928-2904639 | 16 | SLC22A18AS;SLC22A18 | TSS1500;TSS200;exon_2;5UTR | 0.031 | 9.95E-05 |
| chr5 | 139665265-139665716 | 3 | CXXC5 | 5UTR;exon_2 | 0.031 | 1.99E-04 |
| chr6 | 150876798-150876850 | 3 | - | - | 0.031 | 0.001 |
| chr6 | 41784484-41785031 | 4 | PRICKLE4 | exon_5 | 0.03 | 2.58E-05 |
| chr17 | 79919247-79919505 | 5 | LINC01978;LINC01979 | exon_1;exon_5;TSS200 | 0.03 | 0.001 |
| chr12 | 132277231-132277657 | 4 | LOC100130238 | exon_3 | 0.03 | 0.004 |
| chr19 | 40341620-40342071 | 3 | - | - | 0.03 | 0.001 |
| chr20 | 37258014-37258076 | 3 | - | - | 0.03 | 3.22E-04 |
| chr7 | 5351762-5351838 | 3 | TNRC18 | exon_17 | 0.03 | 1.14E-04 |
| chr19 | 56838216-56838728 | 6 | PEG3 | TSS1500 | 0.03 | 0.006 |
| chr13 | 28049444-28049640 | 3 | FLT3 | exon_8;exon_7 | 0.03 | 2.69E-04 |
| chr8 | 143950469-143950771 | 5 | PLEC | exon_1;5UTR | 0.03 | 2.05E-04 |
| chr7 | 113144620-113144862 | 3 | - | - | 0.03 | 6.57E-05 |
| chr1 | 16018041-16018996 | 10 | HSPB7 | exon_1;5UTR;TSS1500;TSS200 | 0.03 | 4.05E-04 |
| chr5 | 55852451-55853146 | 6 | IL31RA | TSS1500 | 0.03 | 1.59E-04 |
| chr19 | 43820595-43821443 | 11 | LYPD5 | TSS200;5UTR;exon_1;TSS1500 | 0.029 | 0.001 |
| chr17 | 49013676-49014159 | 5 | - | - | 0.029 | 0.005 |
| chr2 | 234676972-234678179 | 3 | - | - | 0.029 | 2.13E-05 |
| chr4 | 84496459-84497431 | 6 | - | - | 0.029 | 0.009 |
| chr3 | 170811821-170812169 | 3 | - | - | 0.029 | 3.12E-05 |
| chr6 | 44222512-44222759 | 3 | SLC29A1 | TSS1500 | 0.029 | 0.006 |
| chr22 | 19953572-19953820 | 3 | - | - | 0.029 | 4.83E-04 |
| chr10 | 131675910-131676530 | 6 | - | - | 0.029 | 3.49E-04 |
| chr7 | 100603054-100603694 | 5 | PCOLCE-AS1;PCOLCE | exon_2 | 0.029 | 1.89E-05 |
| chr6 | 30205839-30206484 | 7 | - | - | 0.029 | 0.003 |
| chr20 | 62707925-62708523 | 5 | NTSR1 | TSS1500 | 0.029 | 0.002 |
| chr11 | 128941906-128942567 | 3 | - | - | 0.029 | 0.001 |
| chr20 | 58851076-58853418 | 58 | GNAS;GNAS-AS1 | TSS1500;5UTR;exon_1;TSS200 | 0.029 | 3.23E-05 |
| chr10 | 14972310-14972526 | 4 | MEIG1 | exon_3 | 0.029 | 3.94E-04 |
| chr1 | 15440164-15440434 | 3 | - | - | 0.029 | 0.001 |
| chr22 | 31140445-31141010 | 6 | PLA2G3 | exon_1;5UTR;TSS200;TSS1500 | 0.029 | 0.003 |
| chr2 | 143776205-143776713 | 6 | - | - | 0.029 | 0.001 |
| chr10 | 123150195-123150524 | 3 | HMX2 | exon_2;3UTR | 0.029 | 1.75E-04 |
| chr6 | 28259049-28259704 | 12 | NKAPL;ZKSCAN4 | TSS200;5UTR;exon_1;TSS1500 | 0.029 | 0.003 |
| chr6 | 33804010-33804589 | 8 | MLN | TSS200;TSS1500 | 0.028 | 0.004 |
| chr21 | 42351011-42351555 | 6 | TFF2 | TSS1500;TSS200 | 0.028 | 0.002 |
| chr14 | 95713982-95714510 | 8 | LOC107984703;TCL1A | TSS1500;TSS200;exon_1;5UTR | 0.028 | 0.004 |
| chr12 | 45923074-45923337 | 3 | SCAF11 | exon_13 | 0.028 | 1.59E-05 |
| chr17 | 46841488-46841915 | 3 | - | - | 0.028 | 3.03E-04 |
| chr1 | 153566582-153566928 | 3 | S100A2 | TSS1500 | 0.028 | 0.002 |
| chr19 | 53912237-53912911 | 7 | CACNG7 | 5UTR;exon_2 | 0.028 | 0.001 |
| chr6 | 28922142-28922292 | 3 | - | - | 0.028 | 0.022 |
| chr19 | 39684398-39684603 | 3 | - | - | 0.028 | 9.89E-05 |
| chr1 | 171257563-171258126 | 5 | FMO1 | TSS200;exon_1;exon_2;5UTR;TSS1500 | 0.028 | 3.87E-04 |
| chr16 | 89048068-89048511 | 6 | - | - | 0.028 | 0.002 |
| chr14 | 64842262-64842627 | 3 | - | - | 0.028 | 2.83E-04 |
| chr19 | 18907821-18908097 | 3 | - | - | 0.028 | 5.59E-05 |
| chr2 | 233379977-233380337 | 4 | - | - | 0.028 | 4.34E-04 |
| chr1 | 164576462-164576906 | 6 | - | - | 0.028 | 0.001 |
| chr11 | 556889-557803 | 6 | LMNTD2;LMNTD2-AS1 | exon_8;exon_7;TSS1500 | 0.028 | 0.001 |
| chr10 | 132146882-132147816 | 9 | - | - | 0.027 | 0.003 |
| chr9 | 136052646-136053188 | 4 | - | - | 0.027 | 0.002 |
| chr2 | 232901162-232901578 | 5 | - | - | 0.027 | 0.001 |
| chr16 | 85351168-85351715 | 3 | - | - | 0.027 | 1.63E-05 |
| chr15 | 83285067-83286097 | 13 | BNC1 | TSS1500 | 0.027 | 3.03E-04 |
| chr1 | 203154016-203154561 | 4 | - | - | 0.027 | 4.06E-05 |
| chr5 | 1551756-1552573 | 7 | - | - | 0.027 | 0.003 |
| chr12 | 2999059-2999449 | 3 | - | - | 0.027 | 2.36E-05 |
| chr10 | 113665834-113666140 | 3 | - | - | 0.027 | 1.08E-04 |
| chr17 | 39687338-39687906 | 6 | PGAP3;ERBB2 | exon_1;TSS1500;TSS200 | 0.027 | 0.011 |
| chr1 | 20358686-20359125 | 3 | VWA5B1 | exon_22;3UTR | 0.027 | 7.60E-05 |
| chr12 | 66678989-66679531 | 8 | GRIP1 | TSS200;exon_1;5UTR;TSS1500 | 0.027 | 0.001 |
| chr12 | 130186215-130186317 | 3 | - | - | 0.027 | 2.29E-05 |
| chr20 | 5150860-5151011 | 3 | - | - | 0.027 | 2.69E-04 |
| chr15 | 93900798-93901354 | 4 | LINC01579 | TSS1500;TSS200 | 0.027 | 2.23E-04 |
| chr19 | 2048354-2049404 | 3 | - | - | 0.027 | 1.45E-04 |
| chr6 | 6320819-6320976 | 4 | F13A1 | TSS200;TSS1500 | 0.027 | 0.009 |
| chr18 | 75428245-75428830 | 3 | SMIM21 | TSS1500 | 0.027 | 0.001 |
| chr11 | 78063821-78063998 | 3 | THRSP | exon_1;TSS200 | 0.027 | 0.001 |
| chr12 | 119364725-119365140 | 3 | - | - | 0.027 | 0.001 |
| chr12 | 130069648-130070650 | 8 | LINC02419 | TSS1500 | 0.027 | 0.007 |
| chr2 | 10121557-10121892 | 3 | RRM2 | TSS1500 | 0.027 | 8.20E-05 |
| chr6 | 11779584-11779708 | 3 | ADTRP | TSS1500 | 0.026 | 0.002 |
| chr2 | 136390397-136391168 | 4 | - | - | 0.026 | 4.84E-05 |
| chr17 | 83028991-83029697 | 4 | - | - | 0.026 | 1.80E-04 |
| chr11 | 68010148-68011031 | 8 | ALDH3B1 | TSS200 | 0.026 | 2.77E-04 |
| chr22 | 29204781-29205630 | 4 | EMID1 | TSS1500 | 0.026 | 1.01E-05 |
| chr2 | 218832413-218832597 | 3 | PRKAG3 | TSS1500 | 0.026 | 0.029 |
| chr12 | 52192002-52192851 | 7 | KRT80 | TSS200;TSS1500;exon_1;5UTR | 0.026 | 0.001 |
| chr7 | 157150945-157151183 | 3 | - | - | 0.026 | 0.001 |
| chr5 | 122310858-122311624 | 7 | SNCAIP | TSS1500;TSS200 | 0.026 | 0.002 |
| chr16 | 3022571-3023190 | 4 | HCFC1R1;THOC6 | exon_3;exon_5;exon_4;TSS1500;3UTR | 0.026 | 0.001 |
| chr12 | 6548652-6549459 | 7 | IFFO1 | exon_6;exon_5 | 0.026 | 0.001 |
| chr1 | 2475182-2475356 | 4 | PLCH2 | TSS1500 | 0.026 | 0.001 |
| chr12 | 56996191-56996916 | 4 | GPR182 | exon_2;3UTR | 0.026 | 4.61E-04 |
| chr16 | 31371301-31371815 | 6 | ITGAX | exon_16;exon_17 | 0.026 | 0.001 |
| chr13 | 110870304-110870638 | 5 | PRECSIT | TSS1500;exon_1 | 0.026 | 0.005 |
| chr11 | 13357938-13358465 | 3 | ARNTL | exon_7;exon_8;exon_6;exon_9;5UTR | 0.026 | 2.86E-04 |
| chr2 | 61145091-61145329 | 4 | C2orf74;C2orf74-DT | TSS200;5UTR;exon_1;TSS1500 | 0.026 | 0.003 |
| chr9 | 128733195-128733711 | 11 | ZDHHC12-DT;ZER1 | exon_2;exon_15;exon_14 | 0.025 | 0.015 |
| chr5 | 136058354-136058698 | 3 | - | - | 0.025 | 0.002 |
| chr12 | 64627212-64627756 | 3 | - | - | 0.025 | 1.99E-05 |
| chr8 | 22994487-22995315 | 4 | RHOBTB2 | exon_3;TSS1500 | 0.025 | 2.47E-04 |
| chr1 | 209704596-209705044 | 6 | HSD11B1 | TSS1500;TSS200;exon_2;exon_1 | 0.025 | 3.18E-05 |
| chr10 | 132817093-132817882 | 7 | - | - | 0.025 | 0.001 |
| chr13 | 99991604-99992403 | 9 | - | - | 0.025 | 0.009 |
| chr12 | 121858904-121859502 | 5 | HPD | TSS1500;TSS200;5UTR;exon_3 | 0.025 | 0.001 |
| chr7 | 97021799-97022803 | 6 | DLX5 | exon_2 | 0.025 | 7.35E-05 |
| chr1 | 110870103-110870258 | 3 | CD53 | TSS1500 | 0.025 | 2.13E-05 |
| chr19 | 1112328-1112733 | 3 | - | - | 0.025 | 0.001 |
| chr4 | 150581783-150582075 | 6 | MAB21L2 | TSS1500;TSS200 | 0.025 | 0.001 |
| chr7 | 45921235-45922361 | 28 | IGFBP3 | TSS1500;exon_1;5UTR | 0.024 | 0.014 |
| chr22 | 29745064-29745189 | 3 | - | - | 0.024 | 0.001 |
| chr6 | 82247888-82248607 | 9 | IBTK | TSS200;TSS1500 | 0.024 | 0.001 |
| chr3 | 194369403-194370463 | 9 | LRRC15 | TSS1500;TSS200;5UTR;exon_1 | 0.024 | 0.002 |
| chr6 | 28590229-28590336 | 4 | - | - | 0.024 | 0.006 |
| chr1 | 230714263-230715086 | 7 | AGT | TSS1500;TSS200 | 0.024 | 0.001 |
| chr11 | 6362522-6363187 | 6 | - | - | 0.024 | 3.48E-04 |
| chr1 | 6276179-6276683 | 3 | - | - | 0.024 | 1.30E-04 |
| chr17 | 74417606-74418102 | 3 | - | - | 0.024 | 1.97E-04 |
| chr3 | 57170078-57170769 | 8 | IL17RD | TSS200;TSS1500 | 0.024 | 0.002 |
| chr1 | 201112254-201112558 | 4 | CACNA1S | TSS200;exon_1 | 0.024 | 0.001 |
| chr1 | 1010719-1011963 | 9 | - | - | 0.024 | 2.31E-05 |
| chr5 | 6707651-6708247 | 5 | LINC02102 | TSS1500;TSS200;exon_1 | 0.024 | 1.99E-05 |
| chr1 | 175067267-175068223 | 7 | TNN | 5UTR;exon_1;TSS1500 | 0.024 | 4.29E-04 |
| chr22 | 24494362-24495698 | 17 | ADORA2A-AS1;UPB1 | exon_1;TSS1500;TSS200;5UTR | 0.024 | 0.002 |
| chr18 | 46412551-46412795 | 3 | - | - | 0.024 | 0.001 |
| chr10 | 75043512-75044167 | 5 | DUSP29 | exon_3;exon_4 | 0.024 | 0.001 |
| chr12 | 52248922-52249334 | 5 | KRT7-AS | TSS1500 | 0.024 | 0.001 |
| chr4 | 184808590-184808811 | 3 | ACSL1 | TSS1500;TSS200 | 0.024 | 0.011 |
| chr10 | 97583554-97584407 | 7 | HOGA1;ANKRD2 | TSS1500;exon_8;exon_9;3UTR;TSS200;5UTR;exon_1 | 0.024 | 4.67E-05 |
| chr11 | 117877107-117877521 | 11 | FXYD6;FXYD6-FXYD2 | TSS1500;TSS200;5UTR;exon_1 | 0.024 | 0.006 |
| chr10 | 21494162-21495001 | 6 | MIR1915HG | exon_2 | 0.024 | 9.55E-06 |
| chr12 | 6963113-6963211 | 3 | MIR141;MIR200C;MIR200CHG | TSS1500;TSS200 | 0.024 | 0.001 |
| chr5 | 59356776-59357122 | 4 | PDE4D | TSS200;exon_1 | 0.023 | 0.002 |
| chr17 | 1761825-1762009 | 5 | SERPINF1 | TSS1500;TSS200 | 0.023 | 0.003 |
| chr16 | 69356331-69356528 | 4 | TERF2 | 3UTR;exon_10 | 0.023 | 2.42E-05 |
| chr8 | 66179345-66179847 | 3 | CRH | TSS1500 | 0.023 | 0.005 |
| chr9 | 74887601-74888126 | 9 | TRPM6 | TSS1500;TSS200;exon_1;5UTR | 0.023 | 0.004 |
| chr16 | 81532010-81532149 | 3 | - | - | 0.023 | 2.44E-04 |
| chr6 | 31733234-31733511 | 4 | - | - | 0.023 | 0.002 |
| chr5 | 14410143-14410543 | 4 | - | - | 0.023 | 0.002 |
| chr11 | 115627773-115627980 | 4 | - | - | 0.023 | 1.01E-05 |
| chr16 | 10559160-10559323 | 3 | - | - | 0.023 | 0.002 |
| chr1 | 247315186-247315312 | 3 | - | - | 0.023 | 3.45E-05 |
| chr4 | 106367254-106367556 | 4 | GIMD1 | exon_2 | 0.023 | 1.06E-05 |
| chr11 | 128687370-128688234 | 5 | LOC101929538 | TSS1500 | 0.023 | 1.82E-04 |
| chr10 | 75395618-75395787 | 3 | - | - | 0.023 | 4.56E-04 |
| chr16 | 1954675-1955061 | 7 | RPL3L | exon_1;5UTR;TSS1500;TSS200 | 0.023 | 0.001 |
| chr8 | 144046281-144047097 | 9 | SPATC1 | exon_4;3UTR;exon_5 | 0.023 | 0.007 |
| chr4 | 153781429-153781654 | 3 | SFRP2 | 3UTR;exon_3 | 0.023 | 3.67E-05 |
| chr12 | 52043398-52044434 | 6 | - | - | 0.022 | 0.004 |
| chr3 | 50277626-50278454 | 5 | LSMEM2 | TSS1500 | 0.022 | 0.007 |
| chr16 | 11269642-11270522 | 4 | TNP2 | TSS1500 | 0.022 | 1.63E-05 |
| chr1 | 1330487-1331401 | 8 | TAS1R3 | TSS200;5UTR;exon_1;TSS1500 | 0.022 | 0.001 |
| chr15 | 33067994-33069069 | 12 | FMN1 | exon_1;5UTR;TSS1500;TSS200 | 0.022 | 0.013 |
| chr1 | 155192931-155193414 | 7 | MUC1 | TSS200;TSS1500 | 0.022 | 0.002 |
| chr16 | 85286253-85286550 | 5 | - | - | 0.022 | 0.001 |
| chr9 | 90915884-90916409 | 3 | - | - | 0.022 | 2.62E-04 |
| chr4 | 139891044-139891116 | 3 | - | - | 0.022 | 4.15E-04 |
| chr6 | 28943982-28944389 | 10 | LINC01556 | exon_1 | 0.022 | 0.017 |
| chr1 | 40978906-40979208 | 8 | CTPS1 | TSS1500 | 0.022 | 0.004 |
| chr17 | 35147357-35148012 | 5 | UNC45B | TSS1500;TSS200;5UTR;exon_1 | 0.022 | 0.001 |
| chr8 | 80687249-80687334 | 3 | ZNF704 | exon_4 | 0.022 | 0.002 |
| chr7 | 100102598-100103464 | 5 | MCM7;AP4M1 | TSS1500;exon_5;exon_3;exon_4 | 0.022 | 3.15E-05 |
| chr4 | 83598065-83598361 | 4 | GPAT3 | exon_11;exon_10 | 0.021 | 1.09E-04 |
| chr2 | 74513111-74513619 | 5 | TLX2 | TSS1500 | 0.021 | 0.004 |
| chr20 | 62352866-62352950 | 3 | LAMA5-AS1 | TSS200 | 0.021 | 5.98E-05 |
| chr12 | 53050093-53050178 | 3 | TNS2 | 5UTR;exon_1 | 0.021 | 0.004 |
| chr1 | 18890978-18891718 | 4 | ALDH4A1 | TSS200;TSS1500 | 0.021 | 0.001 |
| chr5 | 110737572-110738594 | 9 | SLC25A46 | TSS1500;TSS200;5UTR;exon_1 | 0.021 | 4.61E-04 |
| chr1 | 37556715-37557393 | 12 | DNALI1 | TSS200;exon_1;5UTR;TSS1500 | 0.021 | 0.007 |
| chr3 | 52834966-52836527 | 10 | MUSTN1 | TSS1500;exon_1;5UTR;TSS200 | 0.021 | 0.001 |
| chr11 | 115128421-115129084 | 3 | - | - | 0.021 | 1.09E-04 |
| chr19 | 48719635-48720588 | 10 | MAMSTR;RASIP1 | TSS1500;3UTR;exon_12;TSS200;5UTR;exon_1 | 0.021 | 0.002 |
| chr12 | 132719638-132720012 | 4 | - | - | 0.021 | 5.10E-05 |
| chr19 | 45781725-45782806 | 6 | DMPK | TSS1500;exon_1;5UTR | 0.021 | 1.16E-04 |
| chr7 | 150955697-150956107 | 9 | KCNH2 | exon_1;5UTR;TSS200;TSS1500 | 0.02 | 0.006 |
| chr6 | 30752030-30752714 | 9 | - | - | 0.02 | 0.002 |
| chr1 | 50047494-50047989 | 7 | ELAVL4 | TSS200;TSS1500 | 0.02 | 2.98E-04 |
| chr19 | 2255420-2256674 | 14 | JSRP1 | TSS200;TSS1500 | 0.02 | 0.003 |
| chr21 | 39451687-39452093 | 6 | SH3BGR | TSS1500;TSS200;5UTR;exon_1 | 0.02 | 0.001 |
| chr21 | 29130410-29131154 | 8 | MAP3K7CL | 5UTR;exon_6;exon_3;exon_2;exon_8;TSS200;exon_1;TSS1500 | 0.02 | 0.001 |
| chr11 | 73646351-73646696 | 3 | PLEKHB1 | TSS1500;TSS200 | 0.02 | 0.007 |
| chr7 | 18086836-18087880 | 8 | HDAC9 | 5UTR;exon_1 | 0.02 | 5.34E-05 |
| chr1 | 111982827-111983142 | 6 | - | - | 0.02 | 0.004 |
| chr7 | 23532992-23533046 | 3 | TRA2A | TSS1500 | 0.02 | 1.37E-06 |
| chr1 | 109622416-109623471 | 5 | AMPD2 | TSS1500 | 0.02 | 0.001 |
| chr12 | 123944342-123944748 | 7 | CCDC92 | 5UTR;exon_3;exon_2;exon_4 | 0.02 | 0.004 |
| chr3 | 189907838-189908123 | 3 | - | - | 0.02 | 2.95E-04 |
| chr1 | 87151004-87152386 | 12 | - | - | 0.02 | 0.002 |
| chr18 | 63476693-63477106 | 8 | SERPINB5 | TSS1500;5UTR;exon_1;TSS200 | 0.02 | 1.50E-04 |
| chr6 | 28988449-28989027 | 18 | HCG15 | exon_3;exon_5 | 0.02 | 0.009 |
| chr6 | 166691113-166691438 | 5 | - | - | 0.019 | 1.17E-04 |
| chr6 | 148972523-148972956 | 3 | - | - | 0.019 | 4.71E-05 |
| chr15 | 49169435-49170463 | 14 | GALK2;MIR4716 | 5UTR;exon_1;TSS1500;TSS200 | 0.019 | 0.003 |
| chr2 | 239483623-239484654 | 5 | - | - | 0.019 | 6.86E-05 |
| chr3 | 77096767-77097076 | 3 | - | - | 0.019 | 2.01E-05 |
| chr16 | 75267464-75268483 | 9 | BCAR1 | TSS1500;TSS200 | 0.019 | 3.87E-04 |
| chr3 | 35678810-35679138 | 5 | ARPP21 | TSS1500;TSS200 | 0.019 | 0.005 |
| chr11 | 72081160-72081634 | 3 | NUMA1 | TSS1500 | 0.019 | 4.95E-04 |
| chr19 | 6463938-6464264 | 10 | CRB3 | 5UTR;exon_1;TSS200;TSS1500 | 0.019 | 0.001 |
| chr15 | 78234771-78235068 | 3 | ACSBG1 | TSS1500 | 0.019 | 0.001 |
| chr1 | 153627003-153627680 | 15 | S100A1;S100A13 | TSS1500;TSS200;5UTR;exon_1;exon_2 | 0.019 | 0.02 |
| chr10 | 49322512-49323193 | 5 | C10orf71 | exon_3;5UTR | 0.019 | 0.002 |
| chr4 | 40195735-40196509 | 5 | RHOH | TSS1500 | 0.019 | 0.001 |
| chr8 | 144503005-144504154 | 12 | GPT | TSS1500;5UTR;exon_1;TSS200;exon_2 | 0.019 | 0.004 |
| chr11 | 125937990-125938344 | 3 | - | - | 0.019 | 7.48E-05 |
| chr12 | 112175752-112176025 | 3 | HECTD4 | exon_66 | 0.019 | 3.64E-04 |
| chr2 | 176099220-176099860 | 10 | HOXD12 | TSS200;TSS1500;exon_1 | 0.019 | 0.003 |
| chr11 | 69703073-69704005 | 8 | FGF19 | exon_1;5UTR;exon_2 | 0.018 | 8.28E-05 |
| chr22 | 25351429-25352200 | 4 | - | - | 0.018 | 1.30E-04 |
| chr1 | 228207538-228208397 | 10 | OBSCN | 5UTR;exon_1;TSS200;TSS1500 | 0.018 | 0.003 |
| chr2 | 11398751-11399163 | 5 | - | - | 0.018 | 3.34E-04 |
| chr8 | 133102811-133103187 | 5 | SLA | TSS1500 | 0.018 | 0.001 |
| chr10 | 84244525-84244948 | 5 | RGR | TSS200;TSS1500 | 0.018 | 3.48E-04 |
| chr20 | 47061826-47062166 | 3 | - | - | 0.018 | 0.001 |
| chr11 | 2898459-2899946 | 18 | SLC22A18;SLC22A18AS | TSS1500;exon_3;TSS200 | 0.018 | 0.001 |
| chr2 | 105438533-105439276 | 9 | FHL2 | TSS1500;TSS200 | 0.018 | 0.003 |
| chr16 | 2212128-2212292 | 4 | PGP;BRICD5 | 3UTR;exon_2;TSS1500 | 0.018 | 3.61E-04 |
| chr3 | 119677879-119678107 | 4 | COX17 | TSS1500 | 0.017 | 3.84E-04 |
| chr5 | 177356737-177357351 | 4 | RGS14 | TSS1500 | 0.017 | 2.69E-05 |
| chr11 | 1870168-1871378 | 10 | LSP1 | 5UTR;exon_1;TSS200;TSS1500 | 0.017 | 0.003 |
| chr13 | 32314886-32315378 | 8 | ZAR1L;BRCA2 | 5UTR;exon_1;TSS200;TSS1500 | 0.017 | 0.001 |
| chr6 | 29627522-29627714 | 5 | GABBR1 | exon_6;exon_1;exon_5 | 0.017 | 0.014 |
| chr7 | 151186916-151188314 | 14 | ASB10 | exon_1;TSS1500;TSS200;5UTR | 0.017 | 0.002 |
| chr17 | 57685083-57685645 | 3 | - | - | 0.017 | 2.17E-05 |
| chr15 | 70094878-70096343 | 10 | TLE3 | TSS1500;TSS200;exon_2 | 0.017 | 0.001 |
| chr16 | 31457989-31458500 | 9 | ARMC5 | exon_1;TSS1500;5UTR;TSS200 | 0.017 | 0.004 |
| chr10 | 84201268-84201891 | 5 | CDHR1 | exon_7 | 0.017 | 4.25E-04 |
| chr2 | 3256805-3257596 | 9 | EIPR1 | exon_5;exon_4 | 0.017 | 0.001 |
| chr1 | 160190316-160190976 | 10 | CASQ1 | TSS200;exon_1;TSS1500 | 0.017 | 0.007 |
| chr6 | 33205441-33205804 | 8 | HSD17B8 | exon_4;exon_5 | 0.017 | 0.005 |
| chr2 | 234171164-234171738 | 5 | - | - | 0.016 | 2.13E-05 |
| chr4 | 103076730-103077340 | 9 | SLC9B2 | TSS200;5UTR;exon_1;TSS1500 | 0.016 | 0.009 |
| chr4 | 52018640-52018906 | 3 | - | - | 0.016 | 1.86E-04 |
| chr16 | 12091155-12091651 | 3 | - | - | 0.016 | 2.61E-04 |
| chr5 | 135578952-135579398 | 11 | SLC25A48;CXCL14 | 5UTR;exon_1;TSS1500;TSS200 | 0.016 | 0.002 |
| chr13 | 113453395-113453697 | 7 | ADPRHL1 | exon_1;5UTR;TSS200;TSS1500 | 0.016 | 0.001 |
| chr2 | 152334098-152334884 | 3 | FMNL2 | TSS1500 | 0.016 | 2.13E-05 |
| chr1 | 3706936-3707490 | 4 | - | - | 0.015 | 0.002 |
| chr1 | 228512185-228512882 | 8 | BTNL10 | exon_2 | 0.015 | 0.001 |
| chr7 | 11832217-11832447 | 7 | THSD7A | TSS200;TSS1500 | 0.015 | 0.002 |
| chr2 | 127025237-127025592 | 3 | - | - | 0.015 | 0.002 |
| chr12 | 131753087-131753244 | 3 | SFSWAP | exon_8 | 0.015 | 4.71E-05 |
| chr21 | 43946702-43946910 | 5 | - | - | 0.015 | 0.001 |
| chr15 | 32673072-32673338 | 5 | SCG5-AS1 | TSS200;TSS1500 | 0.015 | 0.001 |
| chr17 | 81821273-81822171 | 20 | - | - | 0.015 | 0.005 |
| chr1 | 35560593-35561010 | 3 | NCDN | exon_4;exon_3 | 0.014 | 3.23E-04 |
| chr10 | 97571857-97572532 | 5 | ANKRD2 | 5UTR;exon_1;TSS1500;TSS200 | 0.014 | 3.22E-04 |
| chr15 | 99384010-99384075 | 3 | - | - | 0.014 | 6.45E-06 |
| chr4 | 85827621-85827887 | 6 | ARHGAP24 | TSS200;TSS1500 | 0.014 | 0.002 |
| chr8 | 66505505-66505640 | 3 | VXN | exon_3 | 0.014 | 5.10E-05 |
| chr15 | 101055001-101055160 | 4 | LRRK1 | exon_27 | 0.013 | 4.80E-05 |
| chr4 | 88708596-88708993 | 3 | FAM13A-AS1 | TSS1500 | 0.013 | 1.44E-05 |
| chr12 | 114697081-114697801 | 18 | - | - | 0.013 | 0.017 |
| chr16 | 910527-911018 | 3 | LMF1 | exon_5;5UTR;exon_4 | 0.013 | 0.017 |
| chr19 | 48455407-48455786 | 8 | GRWD1;KCNJ14 | exon_7;3UTR;TSS200;5UTR;exon_1 | 0.013 | 0.001 |
| chr1 | 1757111-1757573 | 5 | NADK | exon_6;exon_4;exon_2 | 0.013 | 1.99E-05 |
| chr8 | 133212298-133212647 | 4 | - | - | 0.013 | 8.58E-05 |
| chr14 | 50397288-50397697 | 9 | CDKL1 | TSS1500;5UTR;exon_1;TSS200 | 0.013 | 3.42E-04 |
| chr11 | 1275239-1275593 | 3 | TOLLIP | 3UTR;exon_5;exon_6;exon_3 | 0.013 | 3.16E-04 |
| chr4 | 134201563-134201952 | 7 | PABPC4L | 5UTR;exon_1;TSS200 | 0.012 | 0.007 |
| chr7 | 88306664-88307388 | 10 | STEAP4 | 5UTR;exon_1;TSS200;TSS1500 | 0.012 | 0.002 |
| chr2 | 144691008-144691368 | 3 | - | - | 0.012 | 4.80E-05 |
| chr7 | 73736531-73736721 | 3 | ABHD11 | 3UTR;exon_5;exon_4;exon_6 | 0.012 | 2.45E-05 |
| chr10 | 131956387-131956460 | 3 | PPP2R2D | exon_10;3UTR;exon_9 | 0.012 | 1.09E-04 |
| chr11 | 10801501-10801712 | 3 | SNORD97;EIF4G2 | exon_1;exon_14;TSS200 | 0.012 | 1.72E-04 |
| chr10 | 49679426-49679888 | 9 | C10orf53 | TSS200;5UTR;exon_1;TSS1500 | 0.012 | 0.002 |
| chr17 | 77287349-77288111 | 6 | SEPTIN9 | TSS1500;5UTR;exon_1;TSS200 | 0.012 | 1.68E-04 |
| chr11 | 118887795-118888283 | 6 | - | - | 0.011 | 0.002 |
| chr15 | 99975632-99976155 | 3 | ADAMTS17 | exon_21 | 0.011 | 1.01E-05 |
| chr11 | 120239835-120240178 | 8 | POU2F3 | TSS1500;TSS200;5UTR;exon_1 | 0.011 | 0.001 |
| chr17 | 44170197-44170990 | 9 | ASB16 | TSS1500;exon_1;5UTR;TSS200 | 0.011 | 0.005 |
| chr19 | 48755548-48756195 | 8 | FGF21;FUT1 | 5UTR;exon_2;TSS1500;exon_1;TSS200 | 0.011 | 2.72E-05 |
| chr6 | 52994309-52994577 | 5 | RN7SK | TSS1500 | 0.011 | 0.048 |
| chr1 | 3643004-3643543 | 6 | - | - | 0.011 | 2.85E-05 |
| chr2 | 235534864-235535763 | 8 | - | - | 0.011 | 2.95E-05 |
| chr11 | 120125676-120125759 | 3 | TRIM29 | exon_4 | 0.01 | 0.004 |
| chr6 | 32069320-32069513 | 3 | - | - | 0.01 | 1.86E-04 |
| chr3 | 93979387-93979924 | 5 | ARL13B | TSS1500 | 0.01 | 0.001 |
| chr10 | 119911945-119912128 | 3 | SEC23IP | exon_6;exon_5 | 0.01 | 2.85E-05 |
| chr6 | 28864344-28864895 | 14 | LINC01623 | TSS1500 | 0.009 | 0.005 |
| chr1 | 1914501-1915359 | 11 | CALML6 | TSS1500;TSS200 | 0.009 | 0.006 |
| chr6 | 99607786-99608251 | 5 | - | - | 0.008 | 0.005 |
| chr11 | 36399751-36400044 | 3 | PRR5L | TSS1500 | 0.008 | 2.04E-04 |
| chr6 | 28903599-28903958 | 3 | TRIM27 | 3UTR;exon_8 | 0.008 | 1.17E-04 |
| chr4 | 3317184-3317942 | 7 | RGS12 | exon_2 | 0.007 | 4.67E-05 |
| chr11 | 66852416-66852547 | 3 | PC | exon_14;exon_15;exon_13 | 0.006 | 2.18E-04 |
| chr11 | 45650550-45651005 | 3 | CHST1 | exon_4 | 0.006 | 3.63E-04 |
| chr6 | 52361956-52362462 | 6 | PAQR8 | TSS200 | 0.006 | 0.002 |
| chr17 | 74893685-74893981 | 5 | FADS6 | TSS1500;TSS200 | 0.006 | 0.004 |
| chr11 | 441983-442753 | 8 | ANO9 | TSS200;exon_1;5UTR;TSS1500 | 0.005 | 0.002 |
| chr1 | 228006335-228006775 | 3 | WNT3A | TSS1500 | 0.005 | 0.002 |
| chr11 | 40293068-40293428 | 5 | LRRC4C | TSS1500;exon_1;TSS200 | 0.004 | 1.70E-05 |
| chr12 | 100142567-100143489 | 10 | UHRF1BP1L | TSS200;exon_1;5UTR;TSS1500 | 0.004 | 0.001 |
| chr11 | 106077689-106078379 | 16 | AASDHPPT;KBTBD3 | 5UTR;exon_1;TSS1500 | 0.003 | 3.87E-04 |
| chr6 | 165661326-165662267 | 7 | PDE10A | exon_1;5UTR | 0.003 | 8.41E-05 |
| chr7 | 97731709-97732447 | 10 | TAC1 | 5UTR;exon_1;TSS1500;TSS200 | 0.003 | 0.001 |
| chr7 | 97016677-97016980 | 3 | - | - | 0.002 | 0.001 |
| chr16 | 89422234-89422555 | 4 | - | - | 0.002 | 2.64E-05 |
| chr6 | 31971967-31972690 | 29 | DXO;STK19 | 5UTR;exon_1;exon_2;TSS1500;TSS200;exon_3 | 0.002 | 0.001 |
| chr4 | 76147992-76148420 | 4 | NUP54 | TSS200 | 0.001 | 2.13E-05 |
| chr6 | 29680384-29682030 | 22 | ZFP57 | 5UTR;exon_1;TSS200;TSS1500 | -0.146 | 0.011 |
| chr16 | 86681393-86681804 | 3 | - | - | -0.109 | 0.002 |
| chr5 | 93573282-93574346 | 6 | - | - | -0.08 | 0.009 |
| chr2 | 118848734-118849819 | 9 | EN1 | TSS1500 | -0.072 | 0.04 |
| chr5 | 93595107-93596339 | 8 | - | - | -0.07 | 0.011 |
| chr21 | 29193289-29193317 | 3 | LINC00189 | TSS200;TSS1500 | -0.068 | 0.001 |
| chr14 | 35369207-35370019 | 6 | - | - | -0.064 | 0.001 |
| chr11 | 5596058-5596626 | 8 | TRIM6-TRIM34;TRIM6 | TSS200;TSS1500;5UTR;exon_1 | -0.061 | 0.016 |
| chr16 | 997460-998327 | 8 | - | - | -0.053 | 0.004 |
| chr13 | 112641878-112642133 | 4 | - | - | -0.051 | 0.001 |
| chr3 | 157089590-157089902 | 5 | LINC00881 | TSS200;exon_1;TSS1500 | -0.044 | 0.001 |
| chr6 | 30110855-30111885 | 17 | TRIM31;TRIM31-AS1 | exon_3 | -0.044 | 0.009 |
| chr2 | 118842426-118843680 | 8 | EN1 | 3UTR;exon_2 | -0.043 | 0.016 |
| chr13 | 113323017-113323348 | 4 | LAMP1 | exon_9;3UTR | -0.037 | 4.59E-05 |
| chr22 | 46364065-46364608 | 3 | CELSR1 | exon_33;exon_34 | -0.036 | 0.001 |
| chr20 | 37520365-37520559 | 9 | BLCAP;NNAT | 5UTR;exon_1;TSS1500;TSS200 | -0.03 | 0.018 |
| chr7 | 27103427-27103715 | 4 | HOXA2 | TSS1500 | -0.03 | 0.002 |
| chr6 | 151807883-151808265 | 5 | ESR1 | exon_1;exon_2;exon_3;TSS1500;5UTR | -0.03 | 0.003 |
| chr5 | 139848021-139848477 | 3 | NRG2 | exon_8;exon_10;exon_11 | -0.029 | 0.002 |
| chr16 | 55478812-55479503 | 10 | MMP2 | 5UTR;exon_1;TSS1500;TSS200 | -0.027 | 0.021 |
| chr2 | 241735632-241736040 | 4 | - | - | -0.026 | 0.002 |
| chr8 | 141421202-141421834 | 5 | PTP4A3 | 5UTR;exon_1;exon_2;TSS200 | -0.026 | 0.002 |
| chr1 | 74732898-74733497 | 13 | TYW3;CRYZ | exon_1;TSS1500;5UTR;TSS200 | -0.024 | 0.023 |
| chr19 | 55461424-55461971 | 8 | ISOC2 | TSS1500;TSS200;5UTR;exon_1 | -0.023 | 0.01 |
| chr11 | 75717865-75718101 | 5 | MOGAT2 | 5UTR;exon_1 | -0.018 | 3.68E-04 |
| chr13 | 20232400-20233236 | 10 | GJB6 | TSS1500;TSS200 | -0.017 | 0.004 |
| chr17 | 35578851-35579269 | 4 | PEX12 | TSS1500 | -0.017 | 0.001 |
| chr1 | 43952824-43953111 | 4 | - | - | -0.014 | 0.001 |
| chr18 | 41954455-41954689 | 3 | PIK3C3 | TSS1500 | -0.013 | 0.001 |
| chr10 | 31927982-31928637 | 5 | - | - | -0.012 | 3.34E-05 |
| chr20 | 38033046-38033908 | 7 | RPRD1B;TTI1 | TSS200;TSS1500;5UTR;exon_1 | -0.01 | 0.001 |
| chr2 | 238427463-238427836 | 3 | - | - | -0.006 | 4.25E-05 |
| chr19 | 11481041-11482014 | 7 | ELAVL3 | TSS200;exon_1;5UTR;TSS1500 | -0.004 | 2.56E-04 |
| chr7 | 93890543-93890871 | 8 | TFPI2;TFPI2-DT | exon_1;5UTR;TSS200;TSS1500 | -0.003 | 0.001 |
| chr6 | 31652395-31652583 | 10 | BAG6;APOM | 5UTR;exon_1;TSS1500;TSS200 | -0.002 | 0.007 |
| chr14 | 102592680-102592723 | 4 | RCOR1 | 5UTR;exon_1 | -0.002 | 1.01E-05 |
| ***Handgrip*** |  |  |  |  |  |  |
| chr18 | 58862523-58862646 | 7 | ZNF532 | TSS1500 | -0.004 | 2.85E-06 |
| chr20 | 37520055-37520869 | 28 | BLCAP;NNAT | 5UTR;exon_1;TSS1500;TSS200 | -0.001 | 1.72E-04 |
| ***Perceived disability*** | |  |  |  |  |  |
| chr10 | 133528366-133528714 | 3 | - | - | -0.129 | 0.001 |

Abbreviations: DMR, differentially methylated region; SPPB, Short Physical Performance Battery; 6MWT, 6-Minute Walk Test.

**Supplemental Table S3. Key DMPs/DMRs and their associations with physical function phenotype**

| **DMP/DMR** | **Chromosome** | **Gene** | **Association with physical function phenotype** | **Known GWAS Associations** |
| --- | --- | --- | --- | --- |
| DMP (cg24044084) | chr4 | NEK1 | Hypomethylated with higher SPPB score | Amyotrophic lateral sclerosis / neurodegeneration |
| DMP (cg20660297) | chr12 | KRR1 | Hypomethylated with higher SPPB score | Ribosome assembly / 40S subunit biogenesis |
| DMP (cg17826344) | chr19 | SHANK1 | Hypomethylated with higher SPPB score | Neurodevelopment / synaptic function |
| DMR (chr7: 45921235–45922361) | chr7 | IGFBP3 | Hypermethylated with faster 6MWT walking speed | Muscle mass |
| DMR (chr16: 86578084-86578845) | chr16 | FOXL1 | Hypermethylated with faster 6MWT walking speed | Bone mineral density / osteoporosis |
| DMR (chr19: 44613396-44613597) | chr19 | IGSF23 | Hypermethylated with faster 6MWT walking speed | Bone remodeling and skeletal homeostasis |
| DMR (chr6: 151807883-151808265) | chr6 | ESR1 | Hypomethylated with faster 6MWT walking speed | Bone mineral density / osteoporosis |
| DMP (cg19062112) + DMR (chr14: 102592680-102592723) | chr14 | RCOR1 | Hypomethylated with faster 6MWT walking speed | Neuronal and synaptic gene regulation |
| DMP (cg02867811) | chr6 | RUNX2 | Hypermethylated with higher perceived disability (PAT-D) | Bone mineral density |
| DMP (cg01817084) | chr8 | PPP2R2A | Hypomethylated with better Lifestyle and cardiovascular health (LE8) | Cell growth and DNA damage response |

Abbreviations: DMP, differentially methylated position; DMR, differentially methylated region; LE8: Life’s Essential 8; SPPB, Short Physical Performance Battery; 6MWT, 6-Minute Walk Test.

**References**

1.	Aryee MJ, Jaffe AE, Corrada-Bravo H, Ladd-Acosta C, Feinberg AP, Hansen KD, Irizarry RA. Minfi: a flexible and comprehensive Bioconductor package for the analysis of Infinium DNA methylation microarrays. *Bioinformatics*. 2014;30:1363-1369. doi: 10.1093/bioinformatics/btu049

2.	Pidsley R, Y Wong CC, Volta M, Lunnon K, Mill J, Schalkwyk LC. A data-driven approach to preprocessing Illumina 450K methylation array data. *BMC Genomics*. 2013;14:293. doi: 10.1186/1471-2164-14-293
